# Splicing Characteristics of Dystrophin Pseudoexons and Identification of a Novel Pathogenic Intronic Variant in the *DMD* Gene

**DOI:** 10.3390/genes11101180

**Published:** 2020-10-10

**Authors:** Zhiying Xie, Liuqin Tang, Zhihao Xie, Chengyue Sun, Haoyue Shuai, Chao Zhou, Yilin Liu, Meng Yu, Yiming Zheng, Lingchao Meng, Wei Zhang, Suzanne M. Leal, Zhaoxia Wang, Isabelle Schrauwen, Yun Yuan

**Affiliations:** 1 Department of Neurology, Peking University First Hospital, Beijing 100034, China; xiezhiyingxzy@bjmu.edu.cn (Z.X.); sunchyue@pku.edu.cn (C.S.); Yilin2143@bjmu.edu.cn (Y.L.); carlmryu@163.com (M.Y.); 07447@pkufh.com (Y.Z.); lcmeng@bjmu.edu.cn (L.M.); neurozw@163.com (W.Z.); drwangzx@163.com (Z.W.); 2 Science and Technology, Running Gene Inc., Beijing 100085, China; tangliuqing@running-gene.com (L.T.); zhouchao@running-gene.com (C.Z.); 3 Department of Epidemiology and Biostatistics, West China School of Public Health and West China Fourth Hospital, Sichuan University, Chengdu 610041, China; hlovml@163.com; 4 Center for Statistical Genetics, Sergievsky Center, Taub Institute for Alzheimer’s Disease and the Aging Brain, and the Department of Neurology, Columbia University Medical Center, New York, NY 10032, USA; hs3265@cumc.columbia.edu (H.S.); sml3@cumc.columbia.edu (S.M.L.)

**Keywords:** pseudoexon, canonical exon, splicing characteristics, *DMD*, intronic variants

## Abstract

Pseudoexon (PE) inclusion has been implicated in various dystrophinopathies; however, its splicing characteristics have not been fully investigated. This study aims to analyze the splicing characteristics of dystrophin PEs and compare them with those of dystrophin canonical exons (CEs). Forty-two reported dystrophin PEs were divided into a splice site (ss) group and a splicing regulatory element (SRE) group. Five dystrophin PEs with characteristics of poison exons were identified and categorized as the possible poison exon group. The comparative analysis of each essential splicing signal among different groups of dystrophin PEs and dystrophin CEs revealed that the possible poison exon group had a stronger 3′ ss compared to any other group. As for auxiliary SREs, different groups of dystrophin PEs were found to have a smaller density of diverse types of exonic splicing enhancers and a higher density of several types of exonic splicing silencers compared to dystrophin CEs. In addition, the possible poison exon group had a smaller density of 3′ ss intronic splicing silencers compared to dystrophin CEs. To our knowledge, our findings indicate for the first time that poison exons might exist in *DMD* (the dystrophin gene) and present with different splicing characteristics than other dystrophin PEs and CEs.

## 1. Introduction

The dystrophin or *DMD* (Duchenne muscular dystrophy) gene consists of 79 canonical exons (CEs). Ninety-nine percent of the gene consists of intronic sequences. There are abundant common fragile sites and transposable elements that can cause large and complex genomic rearrangements in *DMD* [1]. Hence, large genomic rearrangements are quite common in *DMD* and account for ~80% of all pathogenic *DMD* variants [2]. The remaining ~20% are mainly small variants involving coding and/or adjacent exon–intron boundaries [2]. Due to recent improvements in sequencing and computational techniques, some previously undetected intronic variants have been increasingly reported as causal for dystrophinopathies [2,3,4]. The accurate splicing of dystrophin pre-mRNA is dependent on the presence and recognition of essential splicing signals, including the donor (5′ ss) and acceptor (3′ ss) splice sites, the branch point (BP) and polypyrimidine tract sequence, and auxiliary *cis*-acting splicing regulatory elements (SREs) [5,6]. Auxiliary SREs are preferentially located near the splice sites and include exonic splicing silencers (ESSs), exonic splicing enhancers (ESEs), intronic splicing silencers (ISSs), and intronic splicing enhancers (ISEs) [6,7]. Those pathogenic intronic variants can cause various non-canonical splicing events of dystrophin pre-mRNA by affecting essential or auxiliary splicing *cis*-elements, including exon-skipping, intron retention, cryptic splice site activation, and pseudoexon (PE)-inclusion [2,3,4,8,9]. Among the aberrant non-canonical splicing events reported in the *DMD* gene, PE inclusion has been frequently described to be involved in the pathogenesis of dystrophinopathies [3,9,10]. The inclusion of dystrophin PEs into the Dp427 m transcript encoding the muscle isoform of dystrophin can lead to absent or reduced expression of dystrophin with varying degrees. This reduced expression of dystrophin ultimately gives rise to Duchenne muscular dystrophy, intermediate muscular dystrophy, or Becker muscular dystrophy (BMD) [9,11].

PEs or cryptic exons are usually located far from the annotated CEs and likely originated from homologous intronic sequences [3]. Without the presence of a pathogenic PE-activating genomic variant (see Methods), a putative PE usually presents a weak exon-like profile. The exon-like profile of a putative PE can be strengthened by the presence of a PE-activating variant, and subsequently, the putative PE will be spliced into the mature transcript with a measurable proportion [3]. A previous study that included 14 dystrophin PEs indicated that, compared to the dystrophin CEs, the dystrophin PEs present with a weaker exon profile in terms of 5′ ss, 3′ ss, ESEs, and ESSs [10]. However, this study did not analyze intronic SREs and included seven PEs without an identified genomic pathogenic PE-activating variant. Therefore, some of the PEs identified from lymphocytes in the previous study could be the products of alternative splicing or the intermediate products of recursive splicing or part of noncoding RNAs [3,10], which can occur in a physiological condition. In another study, the author analyzed the splice sites of 58 dystrophin PEs. This study however did not analyze the characteristics of other splicing signals and included the PEs without a pathogenic PE-activating variant [3]. Hence, the splicing characteristics of dystrophin PEs with a pathogenic PE-activating variant have not been fully investigated. Characterizing these variants and understanding their pathogenic mechanisms are important for the development of genetic therapies, which can induce PE skipping based on the complementary binding of antisense oligonucleotides to splicing motifs.

In this study, we first expand the spectrum of aberrant splicing variants in *DMD* in general by reporting a novel genomic intronic *DMD* variant in a BMD patient, which caused a non-canonical splicing event of dystrophin pre-mRNA (partial intron inclusion). Then, we investigated and further characterized a specific form of aberrant splicing in *DMD*, PE activation, and summarized 42 reported dystrophin PEs from the literature that are activated by a pathogenic PE-activating variant. We analyzed the splicing characteristics of the 42 dystrophin PEs, including both essential splicing signals and auxiliary SREs using several common bioinformatic tools. Furthermore, we compared the splicing characteristics of dystrophin PEs with those of dystrophin CEs.

## 2. Materials and Methods

### 2.1. Patient and mRNA Analysis

This study was approved by the Ethics Committee at Peking University First Hospital. Written informed consent was obtained from the parents for their and their child’s inclusion in the study. A 4.5-year-old boy with clinical features compatible with a BMD phenotype was enrolled. He was presented to Peking University First Hospital at the age of 4.5 years because of an incidental finding of elevation in serum creatine kinase (CK) level (range 3566–6075 IU/L; normal 25–170 IU/L). Physical examination confirmed that he had mild calf hypertrophy but no obvious muscle weakness. Routine genetic testing for myopathies was performed, including a next-generation sequencing-based diagnostic panel [12] covering all exons and flanking regions of genes related to inherited neuromuscular disorders and multiplex ligation-dependent probe amplification (MLPA)-based deletion/duplication analysis of *DMD*. However, this genetic testing did not reveal a causal variant. Next, a diagnostic muscle biopsy was performed. Routine techniques were used for histological, histochemical, and immunohistochemical staining using a panel of primary antibodies against dystrophin (dystrophin-R, DYS1; dystrophin-C, DYS2; dystrophin-N, DYS3; Novocastra Laboratories, Newcastle). Total muscle mRNA was isolated from the remaining muscle tissue using an RNA extraction kit (Invitrogen, La Jolla, CA, USA) and retrotranscribed to cDNA using a HiScript II Q RT SuperMix kit (Vazyme, Nanjing, China). Full length sequences of the entire dystrophin cDNA (NM_004006.2) of the patient were amplified, and Sanger sequencing of 22 overlapping cDNA fragments using primer sets was performed (Table S1) [12].

### 2.2. Dystrophin Pseudoexons

In order to rule out the PEs possibly caused by the alternative splicing or recursive splicing of *DMD* and other unknown conditions that could happen in a physiological condition, we used modified inclusion criteria for dystrophin PEs based on the definition of PE proposed by Dr. Keegan [3]. The modified inclusion criteria include: (1) all or part of a PE sequence is homologous to a tract of *DMD* intronic sequence; (2) the entire sequence of a PE does not overlap with any sequence of the *DMD* CEs; (3) a PE should account for a measurable proportion of the muscle dystrophin mRNA and is observed in patients with dystrophinopathies; (4) a PE without a pathogenic PE-activating variant at genomic level is not included. Thus, a genomic pathogenic *DMD* variant needs to have been identified, which activates a dystrophin PE through the experimentally demonstrated splicing mechanisms, i.e., creation of new splice sites, strengthening cryptic splice sites, creation of new ESEs, and/or disruption of original ESSs. Based on these criteria, we summarized 42 reported dystrophin PEs and their corresponding phenotypes after a thorough search of the literature. Dystrophin PEs and related genetic details were consistently recorded in relation to genomic reference sequence NC_000023.10 (genome build GRCh37/hg19), coding DNA reference sequence NM_004006.2, RNA reference sequence NM_004006.2, and protein reference sequence NP_003997.1 according to the published literature and Human Genome Variation Society nomenclature [13]. Evolutionary constraint of each PE region was estimated by the Genomic Evolutionary Rate Profiling (GERP) score that is calculated based on an alignment of 35 mammalian species [14].

### 2.3. In Silico Prediction

Both essential splicing signals and auxiliary SREs of each dystrophin PE (mutant sequence) and CE were analyzed using several common bioinformatic tools. Splice site consensus motifs of total dystrophin PEs and dystrophin CEs were investigated, as they can be exploited for therapeutic strategies for PE skipping and assist in the understanding of PE inclusion. To construct the 5′ ss and 3′ ss motifs, Multiple Em for Motif Elicitation software (MEME, version 5.1.1) was used [15]. The position weight matrix of each motif was outputted for this study.

#### 2.3.1. Splice Site and Branch Point

The algorithms selected for evaluating the strength of 5′ ss and 3′ ss comprised Human Splicing Finder (HSF Pro from Genomnis) [16], maximum entropy (MaxEnt) [17], first order Markov model (MM), weight matrix model (WMM), and multiple dependence decomposition (MDD) (http://hollywood.mit.edu/burgelab/maxent/Xmaxentscan_scoreseq.html). The BP was predicted using a support vector machine (SVM) learning algorithm, the SVM-BPfinder, of which the output was limited to the AG-dinucleotide exclusion zone only. The BP with the highest score was selected as the best candidate according to the rules specified in SVM-BPfinder [5]. The distance of the BP adenine to the 3′ ss and the pyrimidine content between the BP adenine and the 3′ ss were calculated.

#### 2.3.2. Numbers and Densities of Auxiliary Splicing Regulatory Elements

Exonic SREs were predicted by several bioinformatic prediction tools, which use position-specific scoring matrices to store the target SREs motifs. The HSF tool was used to calculate the number of RESCUE-ESE hexamers [18], FAS-ESS hexamers [19], PESE and PESS octamers [20], exon/intron-identity elements (EIE/IIEs) [7,21], ESE motifs for 9G8 and Tra2-β [16], ESS motif for hnRNP A1 [16], Sironi’s ESS motifs [22], and ESEfinder motifs for SF2/ASF, SF2/ASFB, SC35, SRp40, and SRp55 [23]. EX-SKIP was used to compute the number of neighborhood inference (NI)-ESE or NI-ESS in each dystrophin PE and CE [24]. The density of each ESE or ESS in each exon was obtained by dividing each number by its sequence length. The ratio of total ESEs to total ESSs in each exon was calculated.

As for the intronic SREs, there are significant overlapping patterns between the scoring matrices of computationally predicted ISEs and ISSs motifs [25,26]; therefore, it is inaccurate to functionally classify a published intronic SRE motif as an ISE or an ISS. Hence, the SpliceAid [27], a splicing factor database storing experimentally assessed target RNA sequences, was used to scan and calculate the numbers of ISEs and ISSs in the flanking intronic sequences of each dystrophin PE and CE. A target sequence was classified as an ISS if it was assigned with a positive score by the SpliceAid and as an ISE with a negative score. The density of ISEs or ISSs was calculated for 300 bp intervals flanking each exon, including both downstream (5′ ss) and upstream (3′ ss) ISEs or ISSs. If the flanking intronic sequence of a dystrophin PE or CE is shorter than 300 bp, then only the region up to the neighboring CE is taken. The ratio of total ISSs to total ISEs in each exon was calculated.

### 2.4. Statistical Analysis

The nonparametric Kruskal–Wallis test was used to compare the difference in each splicing signal among different groups of dystrophin PEs and the group of dystrophin CEs. If the Kruskal–Wallis test was statistically significant, the Nemenyi test was used to performed pairwise comparisons to locate the source of significance. The Mann–Whitney U test was employed to compare the difference in each splicing signal and GERP scores between the group of total dystrophin PEs and the group of dystrophin CEs. All tests were two-sided and a *p* value < 0.05 was considered statistically significant using the R software (version 3.1.3; The R Foundation for Statistical Computing, Vienna, Austria; http://www.r-project.org).

## 3. Results

### 3.1. Dystrophin Protein and mRNA Analysis

**Figure 1:**
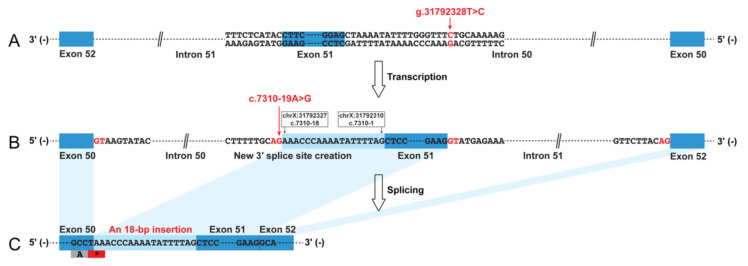
Graphic representation of the non-canonical splicing event caused by a *de novo* and novel genomic intronic variant in the dystrophin (*DMD*) gene. The intronic variant c.7310-19A>G in intron 50 created a new 3′ splice site that was stronger than the natural acceptor site of exon 51. This caused the inclusion of an 18 bp sequences into the mature transcript, which was predicted to create a premature termination codon. (**A**) Patient genome (NC_000023.10); (**B**) dystrophin pre-mRNA; (**C**) dystrophin mRNA (NM_004006.2).

The muscle biopsy revealed a severe reduction in dystrophin-N, a partial reduction in dystrophin-C, and a slight reduction in dystrophin-R (Figure S1), suggesting a molecular diagnosis of BMD in this patient. The dystrophin cDNA analysis identified an insertion of 18 bp sequence into the mature mRNA between exons 50 and 51 (Figure 1 and Figure S1). A BLAT search indicated that the 18 bp sequence was derived from intron 50 (chrX:31792310-31792327). Therefore, this insertion was described as r.7309_7310ins7310-18_7310-1 (NM_004006.2) at mRNA level, which was predicted to create a premature termination codon (PTC), p.(Ser2437Ter), just occurring at the last codon of exon 50. A low residual level of wild-type transcript was present and could be distinguished from the aberrant transcript (Figure S1). Genomic Sanger sequencing subsequently identified a novel variant in intron 50, NC_000023.10:g.31792328T>C (NM_004006.2:c.7310-19A>G). This variant was predicted to create a new 3′ ss (HSF score 91.24; MaxEnt score 11.72) that was stronger than the natural 3′ ss of exon 51 (HSF score 69.98; MaxEnt score 3.73), causing the inclusion of 18 bp intronic sequence into the mature transcript. The genomic variant (c.7310-19A>G) was absent from the dbSNP database (https://www.ncbi.nlm.nih.gov/snp/) and several genomic databases as well, including the Genome Aggregation Database (gnomAD; https://gnomad.broadinstitute.org/), ClinVar (https://www.ncbi.nlm.nih.gov/clinvar/), and Leiden Open Variation Database (LOVD; https://databases.lovd.nl/shared/genes/DMD). The genomic variant (c.7310-19A>G) was a *de novo* variant (Figure S1).

### 3.2. Summary of Dystrophin Pseudoexons

**Figure 2:**
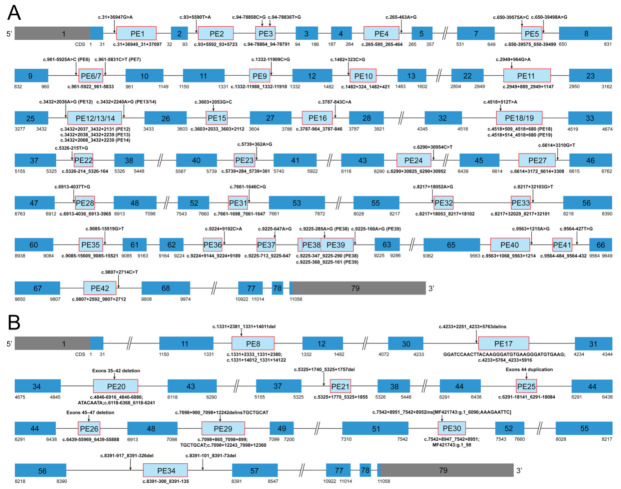
Graphic representation of the forty-two dystrophin pseudoexons. (**A**) Graphic representation of PEs activated by deep intronic single nucleotide variants in *DMD*. (**B**) Graphic representation of PEs introduced by small and large rearrangements in *DMD*. Each PE is shown as a light blue box, and its genomic coordinate is indicated under the light blue box. The PE-activating genomic variant of each PE is shown above an arrow. A dark blue box indicates a canonical exon. The more detailed genetic information about the PEs is provided in Table S2. PE, pseudoexon; ins, insertion; inv, inversion; dup, duplication; del, deletion; delins, deletion-insertion.

Details of the 42 reported dystrophin PEs with pathogenic PE-activating genomic variants in *DMD* are described in Table S2. Graphic representation of the 42 dystrophin PEs is shown in Figure 2. Deep intronic single nucleotide variants (SNVs) were the most commonly reported causes of dystrophin PEs, accounting for 78.57% of the PEs (33/42), through 33 unique pathogenic SNVs. Among the 33 SNVs, 27 SNVs, respectively, activated the inclusion of 27 different PEs (PE1, 2, 4, 6, 7, 9–12, 15, 16, 22–24, 27, 28, 31–33, and 35–42), one SNV activated the inclusion of both PE13 and PE14, one SNV activated the inclusion of both PE18 and PE19, two SNVs (c.[94-78858C>G;94-78836T>G]) combinedly activated the inclusion of PE3, and the remaining two SNVs (c.[650-39575A>C;650-39498A>G]) combinedly activated the inclusion of PE5. One small intronic deletion of an 18 bp sequence (PE21) and eight unique large rearrangements accounted for the causes of the remaining 21.43% dystrophin PEs (9/42). Five of the eight large rearrangements were pure intronic variants and, respectively, activated the inclusion of five different PEs (PE8, 17, 29, 30, and 34), while the remaining three were pathogenic variants involving the exonic and intronic region of *DMD* and, respectively, activated the inclusion of three different PEs (PE20, 25, and 26).

Thirty-one dystrophin PEs (PE1, 2, 4–7, 9, 10, 12–14, 16, 17, 19, 22–24, 27, 28, 30–33, and 35–42) activated by genomic pathogenic *DMD* variants through the creation of new splice sites and/or strengthening cryptic splice sites were categorized as the group of dystrophin PEs with alterations in splice sites (referred to as the “splice site group” from here on). Eleven dystrophin PEs (3, 8, 11, 15, 18, 20, 21, 25, 26, 29, and 34) were activated through the alterations in ESEs and/or ESSs and were categorized as the group of dystrophin PEs with alterations in SREs (referred to as the “SRE group”). Five dystrophin PEs (PE22, 23, 33, 36, and 37) were highly conserved exons that contain a PTC (Figure S2) and therefore were considered to have the characteristics of poison exons. However, we cannot confirm whether these exons are indeed included into the mature mRNA to create a poison exon at any spatiotemporal time point; not without the presence of a pathogenic genomic variant. Thus, these potential poison exons were referred to as the “possible poison exon group” [28].

### 3.3. Comparative Analyses of Essential Splicing Signals

**Figure 3:**
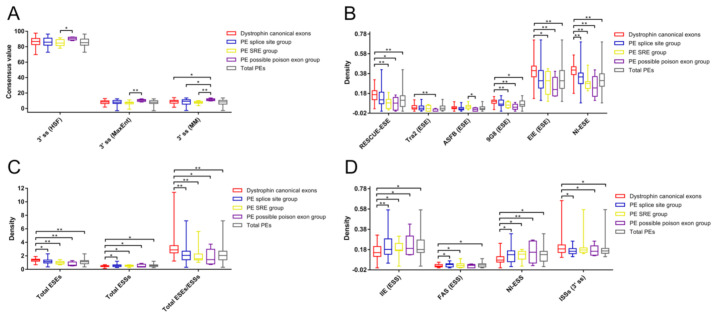
Comparative analyses of different splicing signals among different groups of dystrophin pseudoexons and the group of dystrophin canonical exons. Comparative analysis of the 3′ ss strength (**A**) revealed that the possible poison exon group had a significantly stronger 3′ ss compared to other groups. Comparative analyses of the SREs revealed that different groups of dystrophin PEs had a smaller density of diverse types of ESEs (**B**), a higher density of several types of ESSs (**D**), and a smaller ratio of total ESEs to total ESSs (**C**) compared to the CEs group. Some groups of dystrophin PEs had a smaller density of 3′ ss ISSs compared to the CEs group (**D**). Only splicing signals with significant difference among different groups were included in these figures. Statistics data of other splicing signals are presented in Table S3. Density was calculated as numbers per base pair. Descriptive statistics are presented as box plots, displaying the minimum, first quartile, median, third quartile, and maximum. PE, pseudoexon; SRE, splicing regulatory element; HSF, Human Splicing Finder; MaxEnt, maximum entropy; MM, first order Markov model; ss, splice site; ESE, exonic splicing enhancers; ESS, exonic splicing silencers; ISS, intronic splicing silencers; EIE, exon-identity element; IIE, intron-identity element; NI, neighborhood inference. * *p* < 0.05; ** *p* < 0.001.

The exon length of the possible poison exon group and the total dystrophin PEs group were significantly shorter than that of the dystrophin CEs group (Table S3). In addition, the possible poison exon group had a shorter exon length compared to the SRE group (*p* < 0.001). No statistically significant difference was found between the group of total dystrophin PEs and the group of dystrophin CEs regarding each essential splicing signal. Furthermore, there was no significant difference among different groups of dystrophin PEs and the group of dystrophin CEs in terms of the strength of 5′ ss, the distance of the BP adenine to the 3′ ss, and the pyrimidine content between the BP adenine and the 3′ ss. The only difference was found in the 3′ ss strength among different groups of dystrophin PEs and the group of dystrophin CEs (Table S3; Figure 3A). The possible poison exon group had a significantly stronger 3′ ss predicted by the MM algorithm than the splice site group, the SRE group, and the CEs group. The 3′ ss strength of the possible poison exon group predicted by the HSF or MaxEnt algorithm was also stronger than that of the SRE group.

**Figure 4:**
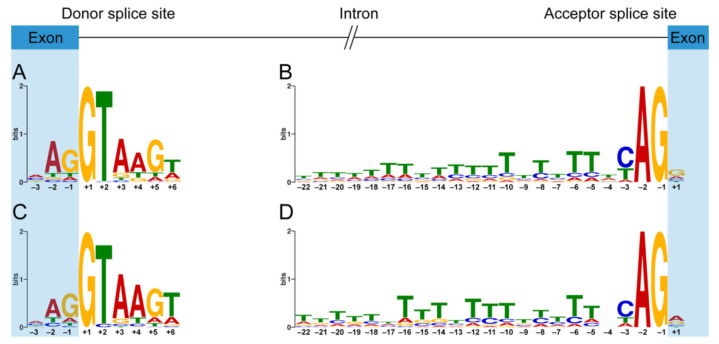
Position weight matrix-based splice site consensus motifs of dystrophin pseudoexons and canonical exons. Sequence logos for donor (**A**) and acceptor (**B**) splice site consensus motifs of dystrophin canonical exons and the consensus sequences are MAG|GTAAGW and TTTWTTTTTTTTTTTTTTWYAG|G, respectively. Sequence logos for donor (**C**) and acceptor (**D**) splice site consensus motifs of dystrophin pseudoexons and the consensus sequences are MAG|GTAAGT and TTTTTHTTTYTTTTYYTTNCAG|R, respectively. The height of each letter reflects the relative frequency of that nucleotide in the respective position. “|” indicates the exon–intron boundary in the consensus sequence. H stands for any nucleotide except G, Y for C or T, N for any nucleotide, R for A or G, M for A or C, and W for A or T.

The 5′ ss and 3′ ss consensus motifs derived from the canonical exon–intron boundaries of *DMD* are similar to the highly conserved canonical motifs derived from the human genome [29]. The position weight matrix-based 5′ ss consensus motif of total dystrophin PEs (Figure 4C) is almost the same as that of dystrophin CEs (Figure 4A). However, there are some minor differences in the 3′ ss consensus motifs between the group of total dystrophin PEs and the group of dystrophin CEs. The nucleotides in positions +1, –4, and –17 of the 3′ ss consensus motif (Figure 4D) derived from total dystrophin PEs appear to be more flexible compared to the canonical one (Figure 4B). Furthermore, 19 of 23 positions of the 3′ ss consensus motif derived from dystrophin PEs have a significantly lower GERP score compared to that of dystrophin CEs (Table S4), indicating that the 3′ ss consensus motif of dystrophin PEs is not as constrained as that of dystrophin CEs.

In addition, among the splice site group, the strength of the 5′ ss group of splice sites that were formed *de novo* or strengthened by a pathogenic genomic variant (*de novo* 5′ ss group) is significantly stronger than that of the cryptic 5′ ss group of splice sites that were only activated as partners of a mutated splice site (cryptic 5′ ss group) (Table S5). The nucleotides in positions –3, +4, and +5 of the 5′ ss consensus motif derived from the cryptic 5′ ss group appear to be more flexible compared to those of the *de novo* 5′ ss group (Figure S3). Although no significant difference in the strength of 3′ ss was observed between the *de novo* 3′ ss group and the cryptic 3′ ss group (Table S5), the nucleotides in positions –21, –19, –17, and –10 of the 3′ ss consensus motifs derived from both the *de novo* 3′ ss group and the cryptic 3′ ss group appear to be flexible (Figure S3). The 5′ ss strength of the *de novo* 5′ ss group is significantly stronger than that of the 5′ ss group of dystrophin canonical exons (canonical 5′ ss group) (Table S6). The 5′ ss consensus motif of the *de novo* 5′ ss group (Figure S3) is almost the same as that of the canonical 5′ ss group (Figure 4A). Although no significant difference in the strength of 3′ ss was observed between the *de novo* 3′ ss group and the canonical 3′ ss group (Table S6), the nucleotides in positions –21, −19, –17,–10, and +1 of the 3′ ss consensus motif derived from the *de novo* 3′ ss group (Figure S3) appear to be more flexible compared to those of the canonical 3′ ss group (Figure 4B).

### 3.4. Comparative Analyses of Auxiliary Splicing Regulatory Elements

Different groups of dystrophin PEs had a smaller density of total ESEs (splice site group, SRE group, and possible poison exon group) compared to the group of dystrophin CEs (Table S3; Figure 3C). The possible poison exon group had a smaller density of RESCUE-ESE, Tra2, 9G8, EIE, or NI-ESE compared to the CEs group (Figure 3B). In addition, the possible poison exon group also had a smaller density of ASFB compared to the SRE group. The ESE density of RESCUE-ESE, 9G8, EIE, or NI-ESE of the SRE group was, respectively, smaller than that of the CEs group. The NI-ESE density of the splice group was smaller than that of the CEs group.

However, different groups of dystrophin PEs had a higher density of total ESSs (splice site group, SRE group, and possible poison exon group) compared to the CEs group (Figure 3C). Both the splice site group and the SRE group had a higher density of IIE, FAS, or NI-ESS compared to the CEs group (Figure 3D). The possible poison exon group also had a higher density of IIE or NI-ESS compared to the CEs group. No significant difference was observed between the splice site group and the SRE group regarding each splicing signal (Table S3).

In general, the group of total dystrophin PEs had a smaller density of total ESEs compared to the group of dystrophin CEs (Table S3; Figure 3C). More specifically, the ESE density of RESCUE-ESE, 9G8, EIE, or NI-ESE of the total dystrophin PEs group was, respectively, smaller than that of the CEs group (Figure 3B). On the contrary, the group of total dystrophin PEs had a higher density of total ESSs compared to the group of dystrophin CEs. The ESS density of IIE, FAS, or NI-ESS of the total dystrophin PEs group was, respectively, higher than that of the CEs group (Figure 3D). Therefore, the total dystrophin PEs group had a smaller ratio of total ESEs to total ESSs compared to the CEs group.

As for the intronic SREs, the significant difference was only observed in the density of 3′ ss ISSs among different groups of dystrophin PEs and the group of dystrophin CEs, where the 3′ ss ISSs density of the splice site group, the possible poison exon group, or the total dystrophin PEs group was smaller than that of the CEs group (Table S3; Figure 3D).

## 4. Discussion

As the majority of human genes consist of multiple exons and introns, the intervening introns must be precisely removed from the pre-mRNA and exons joined together to form a mature mRNA. The pre-mRNA splicing of the *DMD* gene, similar to pre-mRNA splicing of most human genes, is also achieved by the U2-dependent (major) spliceosome [29]. The combinatorial recognition of essential splicing signals that define exon–intron boundaries and auxiliary *cis*-acting SREs is critical for a precise pre-mRNA splicing process [30]. Pathogenic genomic *DMD* variants, especially intronic variants that alter the essential or auxiliary splicing *cis*-elements, can cause splicing errors and result in various non-canonical splicing events [2,3,4,8,9]. PE inclusion, one of the non-canonical splicing events identified in *DMD*, has been reported to be implicated in various dystrophinopathies; however, its splicing characteristics have not been fully investigated [3,9,10]. This study was focused on the further characterization of aberrant splicing in *DMD*. First, we identified a novel intronic DNA variant causing a non-canonical splicing event in *DMD*. Then, we focused on a subset of aberrant splicing that needed further exploration, i.e., PE activation, and analyzed the splicing characteristics of 42 dystrophin PEs and compared them with those of dystrophin CEs.

As the largest gene annotated in the human genome, the *DMD* gene spans over 2.5 Mb and the vast majority of it is intronic sequences [1]. The length of 36 introns in *DMD* are 10× longer than the median intron length of human genes, and three of them are more than 100× the median size [30]. In addition, common fragile sites and repetitive elements that can mediate large genomic mutation events are relatively common in the vast intronic region of *DMD* [31]. The above complex structure of *DMD* makes the occurrence of intronic DNA variants including large intronic rearrangements not uncommon. However, it is difficult to assess the pathogenicity of intronic variants without experimental evidence. It has been well-established that mRNA analysis of dystrophin cDNA can be used to detect non-canonical splicing events in *DMD* and then search for pathogenic genomic variants [4,8,9]. Therefore, we performed mRNA analysis of dystrophin cDNA in a BMD patient after a negative finding in clinical genetic testing. We successfully identified a non-canonical splicing event in *DMD*, an inclusion of 18 bp intronic sequence into the mature mRNA, and then identified a novel intronic DNA variant in intron 50 (c.7310-19A>G). A reduced expressed dystrophin protein was also identified in this patient via immunohistochemical staining. This latter could be due to nonsense-mediated decay (NMD) of the aberrant transcript and/or due to a truncated dystrophin that is vulnerable to degradation, either of which would lead to absence or severe reduction in dystrophin. The mixture of wild-type transcript and aberrant transcript resulted in a partial expression of dystrophin observed in this patient. Based on the phenotype (PP4), genotype (PS2 and PM2), results of dystrophin expression and mRNA analysis (PS3), and in silico predictions (PP3), this genomic variant can be classified as a pathogenic variant according to the American College of Medical Genetics (ACMG) guidelines for the interpretation of sequence variants [32].

The exon definition mechanism involving the combinatorial recognition of essential splicing signals is the first step and probably most important in pre-mRNA splicing [33], which indicates that a splicing event with a predominant occurrence should have a strong exon profile of essential splicing signals. As a disease-related PE-inclusion event occurs in preference over the constitutive splicing of the unaffected CEs occurs in *DMD*, the essential splicing signals strength of dystrophin PEs enhanced by PE-activating genomic variants should not be weaker than that of the dystrophin CEs. Our study found no statistically significant difference between the total dystrophin PEs group and the dystrophin CEs group in terms of each essential splicing signal, suggesting that dystrophin PEs are not weaker than dystrophin CEs regarding the essential splicing signals. These findings are contrary to the findings in a previous study that 14 dystrophin PEs had a weaker exon profile compared to dystrophin CEs in terms of essential splicing signals [10]. The possible reason contributing to the contrary findings is that the previous study included some dystrophin PEs without a pathogenic PE-activating variant. Those PEs without a pathogenic genomic variant are possibly originated from the alternative splicing of *DMD*, which typically present a weak characteristic to the spliceosome. In addition, some of those PEs identified from lymphocytes could be the intermediate products of recursive splicing or part of noncoding RNAs [10]. Note that recursive splicing is important for the splicing of some of the large dystrophin introns [3]. Last, expression levels of wild-type and mutant transcripts associated with these PE inclusions were not available and were therefore not taken into account into the analysis.

We also analyzed the splicing characteristics of possible poison exons in *DMD*. Poison exons are alternative exons with high-level conservation and contain a PTC. Inclusion of a poison exon in a mature transcript causes NMD and decreases the expression of the resulting protein [28]. Poison exons show a dynamic spatiotemporal expression profile and have been found important during development by altering gene expression via alternative splicing [34]. Recently, poison exons have also been shown to be relevant to Mendelian diseases, as pathogenic genomic variants that promote the inclusion of these exons into the mature mRNA have been successfully identified in certain Mendelian diseases [28,34]. In the current study, we identified five dystrophin Pes, which are highly conserved and contain a PTC. These were considered as potential poison exons in *DMD*. As we have no further confirmation of expression of these potential poison exons without the presence of a pathogenic variant, these possible poison exons are awaiting to be proved as alternative and therefore bona fide poison exons in *DMD*. Analysis of essential splicing signals revealed that the mutant possible poison exon group has a stronger 3′ ss compared to any other group. The 3′ ss strength of the possible poison exons was strengthened by the presence of pathogenic *DMD* variants at certain positions of 3′ ss. This study shows, to our knowledge, for the first time that several possible poison exons may exist in the *DMD* gene, which were found to cause dystrophinopathies by promoting poison exon inclusion. Hence, genomic sequencing of the intronic regions around these possible poison exons should be considered in unsolved dystrophinopathies after routine genetic testing.

It is well-known that the presence of essential splicing signals is necessary but not sufficient to the accurate splicing of pre-mRNA [5,7]. Other auxiliary SREs are not only important in regulation of splicing but are also crucial for splice site recognition [6,7,35]. The main significant differences were found in exonic SREs among different groups of dystrophin PEs and the group of dystrophin CEs. Compared to the CEs group, different groups of dystrophin PEs have a smaller density of diverse types of ESEs, but on the contrary, they have a higher density of several types of ESSs, which is similar to the findings in a previous study, which included 14 dystrophin PEs [10]. With regard to the intronic SREs, the only significant difference is that the 3′ ss ISSs density of dystrophin PEs is smaller than that of dystrophin CEs. The ESEs and ISSs enriched in exons and introns can facilitate exon definition, whereas ESSs and ISEs can facilitate intron definition [6,7,27,35,36]. From the perspective of auxiliary SREs, an exon presenting a strong exon profile means that it has a high occurrence of ESEs and/or ISSs and a low occurrence of ESSs and/or ISEs. Hence, the differences in exonic and intronic SREs between dystrophin PEs and CEs observed in our study indicate that dystrophin PEs present with a weaker exon profile compared to dystrophin CEs in terms of auxiliary SREs. As poison exons are also conserved between species in addition to CEs [28,37], the difference in the presence of auxiliary SREs between poison exons and CEs might be due to the different function of these exons. Wild-type poison exons are usually only expressed in very specific times during development, with restriction to certain cell types [34]. As a very limited number of poison exons have been identified, their splicing characteristics have not been well investigated. Therefore, although our study is small, we provide some evidence that poison exons may show different characteristics in splicing signals regulating their expression.

In conclusion, we identified a novel intronic DNA variant (c.7310-19A>G in intron 50) that caused a non-canonical splicing event in *DMD* (partial intron inclusion), expanding the genetic spectrum of aberrant splicing in dystrophinopathies in general. Additionally, we investigated a specific subset of aberrant splicing, PE inclusion, and provide evidence that dystrophin PEs present a weaker exon profile compared to dystrophin CEs in terms of auxiliary SREs. Last, we highlight for the first time that several possible poison exons may exist in *DMD* and may be implicated in dystrophinopathies. These possible poison exons also show different splicing characteristics than other dystrophin PEs and CEs, which may be consistent with their different function in gene expression and the regulation thereof. Overall, our paper characterizes and increases knowledge on various aberrant splicing mechanisms in *DMD*.

## Supplementary Materials

The following are available online at https://www.mdpi.com/2073-4425/11/10/1180/s1, Figure S1. Pathological changes of the patient and Sanger sequencing of the aberrant transcript and the genomic variant in the *DMD* gene. (**A**) and (**E**) hematoxylin and eosin staining (×20); (**B**) and (**F**) immunohistochemical staining for dystrophin-N (×20); (**C**) and (**G**) dystrophin-C (×20); (**D**) and (**H**) dystrophin-R (×20); (**A**)–(**D**), a healthy control; (**E**)–(**H**), the patient. (**I**) Diagrammatic representation of the dystrophin protein showing four main domains and the positions of the epitopes of the dystrophin-N (amino-terminal), dystrophin-C (carboxyl-terminal), and dystrophin-R (central rod) antibodies. The obvious or partial expression of dystrophin-C regardless of the expression of dystrophin-N and dystrophin-R indicates a molecular diagnosis of Becker muscular dystrophy. (**J**) Sanger sequencing of the aberrant transcript of *DMD* (NM_004006.2) revealed an insertion of 18 bp sequence originating from intron 50 into the mature mRNA between exons 50 and 51. Two overlapping sequences can be recognized following the sequence of exon 51; they can be distinguished into the sequence of exon 50 and the inserted sequence. (**K**) Sanger sequencing of the genomic DNA derived from peripheral blood sample corresponding to the area surrounding the inserted sequence revealed a single-base substitution, g.31792328T>C (c.7310-19A>G), adjacent to the insertion. (**L**) Pedigree of the patient’s family. As shown in the pedigree chart, the patient (II:1) had the genomic *DMD* variant (c.7310-19A>G), whereas his parents (unaffected individuals, I:1 and I:2) did not have the variant. Figure S2. The high-level conservation of five dystrophin pseudoexons. Five dystrophin pseudoexons (PEs) are conserved across representative placental mammals, including human, marmoset, mouse, ferret, and dolphin. The high-level conservation of each PE is also illustrated by the Genomic Evolutionary Rate Profiling (GERP) score across the PE region. GERP scores are calculated based on an alignment of 35 mammalian species. A positive GERP score indicates that a site is probably under evolutionary constraint, whereas a negative GERP score indicates that a site is probably evolving neutrally. A GERP score above the threshold of 2 indicates a highly conserved site. These figures were modified from the UCSC Browser. (**A**) PE22 (median GERP score 2.63, range −6.23–5.58); (**B**) PE23 (median GERP score 4.69, range −0.60–5.70); (**C**) PE33 (median GERP score 2.20, range −7.65–4.38); (**D**) PE36 (median GERP score 2.07, range −8.81–4.63); (**E**) PE37 (median GERP score 3.19, range −5.80–5.00). Figure S3. Position weight matrix-based splice site consensus motifs of different splice site groups. Sequence logos for (**A**) the 5′ ss group that were formed *de novo* or strengthened by a pathogenic variant (*de novo* 5′ ss group) and (**C**) the cryptic 5′ ss group that were only activated as partners of a mutated splice site (cryptic 5′ ss group) and the consensus sequences are MAG|GTAAGT and NAG|GTAMKT, respectively. Sequence logos for (**B**) the *de novo* 3′ ss group and (**D**) the cryptic 3′ ss group and the consensus sequences are WNTNYHTTTTTTNTYYYTAYAG|R and TKTKTNTTTTTTWTYTYTBYAG|R, respectively. The height of each letter reflects the relative frequency of that nucleotide in the respective position. “|” indicates the exon–intron boundary in the consensus sequence. N stands for any nucleotide, H for any nucleotide except G, B for any nucleotide except A, Y for C or T, R for A or G, M for A or C, K for G or T, and W for A or T. Table S1. The list of primers used for the *DMD* gene cDNA sequence amplification. Table S2. Details of forty-two reported pseudoexons in the *DMD* gene. Table S3. Statistics data of each essential splicing signal and splicing regulatory element of different groups of dystrophin pseudoexons and the group of dystrophin canonical exons. Table S4. Comparative analysis of GERP score of each position in the acceptor splice site consensus motif between dystrophin canonical exons and pseudoexons. Table S5. Comparative analyses of essential splicing signals between different splice site groups. Table S6. Comparative analyses of essential splicing signals between *de novo* and canonical splice site groups.
