# Supplementary material for: Splicing Characteristics of Dystrophin Pseudoexons and Identification of a Novel Pathogenic Intronic Variant in the DMD Gene
Source: Genes (Basel). 2020 Oct 10;11(10):1180. doi: 10.3390/genes11101180 (PMC7650627; doi:10.3390/genes11101180)
Supplement: Supplementary file 1 [file genes-11-01180-s001.zip › Supplementary files/Table S2.pdf]

**Table S2. Details of forty-two reported pseudoexons in the *DMD* gene.**

| Number | Origin intron | Size (bp) | Pseudoexons | | Pathogenic variant (DNA level description) | | | RNA level description | Protein level (Effect on ORF) | Phenotype | Reference |
|---|---|---|---|---|---|---|---|---|---|---|---|
| | | | PE Sequence | PE genomic position (c. description) | Variant type | c. description | g. description | r. description | | | |
| 1 | 1 | 149 | CTTCTCGAGTTCATAGGAGACTTTCAGTTTCCAGTGACCTGGAAACTCACCATTCCTCATCACCATCCTTTACTGTAGTAACTTCCTTTTACCTGACCACCCTGCATAGTCACAGAAGATGCACTCCTGACAAGTGATCCTCAAAACAG | c.31+36949_31+37097 | Intronic SNV | c.31+36947G>A | g.33192452C>T | r.[=,31_32ins31+36949_31+37097] | Out-of-frame | BMD | [1,2] |
| 2 | 2 | 132 | GTTGAATCTGTTCCTGCAGCAACTAGTAACCCCAACAATCTGACTGGTGAGAAATTCAATCTCCGTAACATTTTGCATTCTTCTACTTCTTCCCACTTCCATTCCAAAGGGCCAGGTAGAGTCCAGAATACT | c.93+5592_93+5723 | Intronic SNV | c.93+5590T>A | g.33032666A>T | r.93_94ins93+5592_93+5723 | In-frame | BMD | [3,4] |
| 3 | 2 | 74 | AAAGAA(C>G)TCTCAATTGCTTTACTCTGAA(T>G)AAGATAATACTGTTTAGAAGACAAATCATTGCAAGTTTTACATCT | c.94-78864_94-78791 | Intronic SNV | c.[94-78858C>G;94-78836T>G] | g.[32946773A>C;32946795G>C] | r.93_94ins[aaagaag;94-78857_94-78837;g;94-78835_94-78791] | Out-of-frame | NA | [5] |
| 4 | 4 | 132 | ATGCTTGTGTTTAACTTTACTCCACCTTAAACATTTGAGGAGTGTGAAGGACAGGAGACACAGAGATTTGCCTTGATTAAGGCAAATAAAACCCTGCCAGATTTTCATTTCCAAACACAGTCCTAGACAGAG | c.265-595_265-464 | Intronic SNV | c.265-463A>G | g.32841967T>C | r.264_265ins265-595_265-464 | Out-of-frame | DMD | [6] |
| 5 | 7 | 77 | (A>C)GTGAATTTCACACCTCTCCTTTTGAAAGATTCATTTCTATGAATTTGGGACAGCTTCCTAGTATGATATTCCATCT | c.650-39575_650-39499 | Intronic SNV | c.[650-39575A>C;650-39498A>G] | g.[32756908T>C;32756985T>G] | r.[649_650ins650-39575_650-39499,=] | Out-of-frame | BMD | [7] |
| 6 | 9 | 90 | AGATTGATCATATTGGGATAAAGACGTGTTTTGGAATCCAAACAACCTGGTTTTAAGTCCCAGAACCACCAGTTACCTTTGTGACCTTTG | c.961-5922_961-5833 | Intronic SNV | c.961-5925A>C | g.32669194T>G | r.[960_961ins961-5922_961-5833,=] | Out-of-frame | BMD | [8] |
| 7 | 9 | 90 | AGATTGATCATATTGGGATAAAGACGTGTTTTGGAATCCAAACAACCTGGTTTTAAGTCCCAGAACCACCAGTTACCTTTGTGACCTTTG | c.961-5922_961-5833 | Intronic SNV | c.961-5831C>T | g.32669100G>A | r.[=,960_961ins961-5922_961-5833] | Out-of-frame | BMD | [1,2] |
| 8 | 11 | 159 | ACAGGGTTTGGATAGATCCAGTCGGAAGCCATTATTTCCTGCCTAAGCCAAACCTTTTCTACAAGAAATGGTAAAGGGCGTTCTTCAATCTTAAAGAAAAGGATGTTAATGAGCAATGAGTCATCATCTGAAGTAACAAAACTCACTGGTGATAGTAAG | c.1331+2333_1331+2380; c.1331+14012_1331+14122 | Large intronic deletion | c.1331+2381_1331+14011del | g.32648238_32659868del | r.[1331_1332ins[1331+2333_1331+2380;1331+14012_1331+14122],=]/r.[=,1331_1332insY13186.2:g.2333_2491] | Out-of-frame | XLDCM | [9–11] |
| 9 | 11 | 79 | GCTGGAGTGAAGTGGTGTGATCTCAGCTCACTGCAACCTCTGTCCCCCCGGGTTCAAGTGATTCTCCTGCCTCAGCCTC | c.1332-11988_1332-11910 | Intronic SNV | c.1332-11909C>G | g.32644479G>C | r.[=,1331_1332ins1332-11988_1332-11910] | Out-of-frame | BMD | [12] |
| 10 | 12 | 98 | CATGCACCACCATGTGAAAACTCTGTGAAAAGGCCCTCACCAGATGCTAACATCTTGATCTTGGATTTCCCAAACTCGAGAACTGTGAAAAAATAAAG | c.1482+324_1482+421 | Intronic SNV | c.1482+323C>G | g.32632097G>C | r.[=,1482_1483ins1482+324_1482+421] | Out-of-frame | BMD | [13] |
| 11 | 22 | 259 | ACGGAGTTTCATTCTTGTTGCCCAGGCTGGAGTGCAGCAGTGCTATCTCGGCTCACTGCAACCTACGCCTCCCGG(G>A)TTCAAGTGATTCTCCTGCCTTAGCCTCCTGAGTAGCTGGGATTATAGTGCGTGCCATCACGCCTGGCTAATTTTTGTATTTTTAAAGTAAAGATGGGGTTTCACCATCTTGGCCATGCTGGTCTCAAACTCCTGACCTCAGATGATCTGCCTGCCTCAGCCTCCCAAAGTGCTGGGATTACAG | c.2949+889_2949+1147 | Intronic SNV | c.2949+964G>A | g.32489317C>T | r.2949 2950ins2949+889_2949+1147 | Out-of-frame | DMD | [14] |
| 12 | 25 | 95 | GTATCACTCTGGCCATGTTCTGACTTTGTAGCCAAATGAGTTAGGTTGTAAAAGGAAGGAACAATGGCGCTCAAGGAGAAGAAGAAGACGATGCG | c.3432+2037_3432+2131 | Intronic SNV | c.3432+2036A>G | g.32479520T>C | r.[=,3432_3433ins3432+2037_3432+2131] | Out-of-frame | BMD | [15] |
| 13 | 25 | 202 | TATCACTCTGGCCATGTTCTGACTTTGTAGCCAAATGAGTTAGGTTGTAAAAGGAAGGAACAATGGCGCTCAAGGAGAAGAAGAAGACGATGCGGTAAAAACAAGGAAGCCATATGTGAATATTGTTACCAATTCAGCATTCCAGAGAGAATAATGGAAATGAAGTGTAAATCTATGCATTACAGAAATATCTACAGACAAA | c.3432+2038_3432+2239 | Intronic SNV | c.3432+2240A>G | g.32479316T>C | r.3432_3433ins3432+2038_3432+2239 | Out-of-frame | DMD | [16,17] |
| 14 | 25 | 172 | CCAAATGAGTTAGGTTGTAAAAGGAAGGAACAATGGCGCTCAAGGAGAAGAAGAAGACGATGCGGTAAAAACAAGGAAGCCATATGTGAA | c.3432+2068_3432+2239 | Intronic SNV | c.3432+2240A>G | g.32479316T>C | r.3432_3433ins3432+2068_3432+2239 | Out-of-frame | DMD | [16] |

| | | | | | | | | | | | |
|---|---|---|---|---|---|---|---|---|---|---|---|
| | | | TATTGTTACCAATTCAGCATTCCAGAGAGAATAATGGAAATGAAGTGTAAATCTATGCATTACAGAAATATCTACAGACAAA | | | | | | | | |
| 15 | 26 | 80 | AAAACTGCAATTCCCAGATA(G>C)GTCAAATGATTTAGCCATAGTCACAGACTTTATTTGTGGTAGAGCCCACAGGATTGAAG | c.3603+2033_3603+2112 | Intronic SNV | c.3603+2053G>C | g.32470726C>G | r.[3603_3604ins3603+2033_3603+2112,=] | Out-of-frame | BMD | [18] |
| 16 | 27 | 119 | ATGTTGCAGTTGTTTTCTTCTATTTTGAGTCTGTTTTTCAATTACTTGATGGTGTCCTTTGATGAATAGAAGGTCCTAATTTTAATGTAGTCGAATGTATTCATCTCTTTTTTCTTACG | c.3787-964_3787-846 | Intronic SNV | c.3787-843C>A | g.32460274G>T | r.3786_3787ins3787-964_3787-846 | Out-of-frame | DMD | [3] |
| 17 | 30 | 189 | GGATCCAACTTACAAGGGATGTGAAGGGATGTGAAGGGAATCCAACTTACAAGGGATGTGAAGGACCTCTTCGAGGAGAACTACAAACCACTGCTCAATGAAATAAAAGAGGATACAAACAAATGGAAGAACATTCCATGCTCATGGGTAGGAAGTGTCAATATCGTGAAAATGGCCATACTGCCCAAG | GGATCCAACTTACAAGGGATGTGAAGGGATGTGAAG; c.4233+5764_4233+5916 | Large intronic deletion-insertion | c.4233+2251_4233+5763delinsCAAGGGATCCAACTTACAAGGGATGTGAAGGGATGTGAAG | g.32424106_32427618delinsCTTCACATCCCTTCACATCCCTTGTAAGTTGGATCCCTTG | r.[=,4233_4234ins[ggauccaacuuacaagggaugugaagggaugugaag;4233+5764_4233+5916]] | Out-of-frame | BMD | [13] |
| 18 | 32 | 172 | TACAGGATATACAGTGAAATGCCTCAAGACAACACCGAATTAAAAGGCATGGAAAGGTACAAATAGCCTAAAGTATAAATCTGATTTAAACTTTATAATATTGAACGGTGTCCTATGTTGCTGTTATTGGAAACTCACCAACTGAGATGATTCCTCTGAAAACAAAGCTGAG | c.4518+509_4518+680 | Intronic SNV | c.4518+512T>A | g.32407106A>T | r.4518_4519ins[uaca;4518+513_4518+680] | Out-of-frame | DMD | [19] |
| 19 | 32 | 167 | GATATACAGTGAAATGCCTCAAGACAACACCGAATTAAAAGGCATGGAAAGGTACAAATAGCCTAAAGTATAAATCTGATTTAAACTTTATAATATTGAACGGTGTCCTATGTTGCTGTTATTGGAAACTCACCAACTGAGATGATTCCTCTGAAAACAAAGCTGAG | c.4518+514_4518+680 | Intronic SNV | c.4518+512T>A | g.32407106A>T | r.4518_4519ins4518+514_4518+680 | Out-of-frame | DMD | [19] |
| 20 | 34 and 42 | 167 | ATGTTCCCGTTTTATAGATGAACAAATACAAATACAATACAGCCAAGAAGATATGTTGGTGCACGTTTCTGGTACCTGACCTAATCAGGCTAGCGGAGTAGCCCTAAACATTCCACCCAAGACTCCAGGCTTGGAGCCATCAGAAGATGGTAGTAAAATTCTACCAG | c.4846-6916_4846-6886; ATACAATA; c.6118-6368_6118-6241 | Large rearrangement involving exonic and intronic region (exons 35–42 deletion) | c.[4846-6900_4846-6899insATGTTCCCGTTTTATAG;4846-6885_6118-6369delinsATACAATA] | g.[32312187_32390201delinsTATTGTAT;32390215_32390216insCTATAAAACGGGAACAT] | r.4846_6117delins[4846-6916_4846-6886;auacaaua;6118-6368_6118-6241] | Out-of-frame | DMD | [20] |
| 21 | 37 | 77 | ATGTGACAGACCCAGCCAATACAAGTTTGTGACCAAGACAAGTTTGTGAGTTTTCATTTCACATTCTGCATGAATTT | c.5325+1779_5325+1855 | Small intronic deletion | c.5325+1740_5325+1757del | g.32379148_32379165del | r.5325_5326ins5325+1779_5325+1855 | Out-of-frame | DMD | [21] |
| 22 | 37 | 51 | GTATGTTCAGCTCTGGTGATGTGAAATGTTTCTCCTTATTTGCATCCTCAG | c.5326-214_5326-164 | Intronic SNV | c.5326-215T>G | g.32366860A>C | r.5325_5326ins5327-214_5326-164 | Out-of-frame | DMD | [22] |
| 23 | 40 | 78 | ATGAACTAATGACTTATCGGTATATGGACGACTTCTTACTCATGTTAGCCCATTCATTTCATCAGAGCATCTTCACAC | c.5739+284_5739+361 | Intronic SNV | c.5739+362A>G | g.32360889T>C | r.5739_5740ins5739+284_5739+361 | Out-of-frame | DMD | [5] |
| 24 | 43 | 128 | GCCTGGCTTACAAGAGCTCCTGAAGGAATCACTAAACATGGAAAGGAAAAACCGGTACCAGCCACTGAGAGAAACATACCAAATTGTAAAGACCATCGACCCTATGAAGAAACTGCCTCAACTAACAG | c.6290+30825_6290+30952 | Intronic SNV | c.6290+30954C>T | g.32274692G>A | r.[6290_6291ins6290+30825_6290+30952,=] | Out-of-frame | BMD | [23] |
| 25 | 43 | 58 | ATGGGACCATCTAGCTGCAGGAAAGGAAGCTCAAGGCTCCCACTGAATCTACATTATG | c.6291-18141_6291-18084 | Large rearrangement involving exonic and intronic region (exon 44 duplication) | c.[6291-21015_6438+98743dupinsA;6291-21008_6291-21007insCTCCCCTGAACATGG] | g.[32256187_32256188insCCATGTTCAGGGGAG;32136290_32256195dupinsA] | r.6438_6439ins[6291-18141_6291-18084;6291_6438] | Out-of-frame | NA | [20] |
| 26 | 44 | 82 | AGGGAGTTTCGCTCTTTCGCCCAGGCTGGAGTGAGGTGGCGCAATCTCAGCTGACTGTAACCTCTGCCCCCACCAGGTTCGA | c.6439-55969_6439-55888 | Large rearrangement involving exonic and intronic region (exons 45–47 deletion) | c.6439-55885_6912+26363del | g.31921350_32042516del | r.6439_6912delins6439-55969_6439-55888 | Out-of-frame | DMD | [24] |
| 27 | 45 | 137 | GAGAAGACATACCAGTCGAGGGGTTCTGGGGAGCCAGGCCTTCAAGCAATGGATTGCTGACAACATAATGAAGAGGATTTACTTAGAAT | c.6614+3172_6614+3308 | Intronic SNV | c.6614+3310G>T | g.31983146C>A | r.[6614_6615ins6614+3172_6614+3308] | Out-of-frame | DMD | [12] |

| | | | | | | | | | | | |
|---|---|---|---|---|---|---|---|---|---|---|---|
| | | | AATGTCAGTTGATAAAAGTTTGAATGGGAGACGGAAGCAAGGCAGTG | | | | | | | | |
| 28 | 47 | 72 | GTCAAGGGTGGAGCCAGGTGCAGATAATTGAATCATGGAAGAGGATCCCCCATACTGTTCTCCTGATAATCA | c.6913-4036_6913-3965 | Intronic SNV | c.6913-4037T>G | g.31897527A>C | r.6912_6913ins6913-4036_6913-3965 | Out-of-frame | DMD | [12,25] |
| 29 | 48 | 161 | CAATCTCCCCGTATAGTCTGGCCACATCAAATTACTGCTGCATTGGCCTGACGTAACTGCTGCATTGTACCCGGATGGCCTCTTTCTCGATCTTCAGAGGTACTTGTCTTCTGGCCGGCTCTCTTCCTGCTTCTGCTATTTCGCCGGTGCATGCTGCTCAG | c.7098+865_7098+899; TGCTGCAT; c.7098+12243_7098+12360 | Large intronic deletion-insertion | c.7098+900_7098+12242delinsTGCTGCAT | g.31881063_31892405delinsATGCAGCA | r.7098_7099ins[7098+865_7098+899;ugcugcau;7098+12243_7098+12360] | Out-of-frame | DMD | [13] |
| 30 | 51 | 103 | AATTCGGAGGAGGAGCCAAGATGGCCGAATAGGAACAGCTCCGGTCTACAGCTCCCAGCGTGAGCGACGCAGAAGACGGGTGATTTCTGCATTTCCATCTGAG | c.7542+8947_7542+8951; MF421743:g.1_98 | Large intronic insertion (MF421743; LINE-1 insertion) | c.7542+8951_7542+8952ins[MF421743:g.1_6096;AAAGAATTC] | g.31783125_31783126ins[MF421743:g.1_6096inv;GAATTCTTT] | r.[7542_7543ins[7542+8947_7542+8951;MF421743:g.1_98],=] | Out-of-frame | BMD | [26] |
| 31 | 52 | 52 | ATAAACCTCTCAGATTATCTAGGCAAGATCATAGAAGAACACGTATGTCCAG | c.7661-1698_7661-1647 | Intronic SNV | c.7661-1646C>G | g.31699349G>C | r.[=,7660_7661ins7661-1698_7661-1647] | Out-of-frame | BMD | [13] |
| 32 | 55 | 50 | AAATGGAACACCACCAGAAAAACAAGAATTTGAAAGACGAGATGAGAAAA | c.8217+18053_8217+18102 | Intronic SNV | c.8217+18052A>G | g.31627738T>C | r.8217_8218ins8217+18053_8217+18102 | Out-of-frame | DMD | [21] |
| 33 | 55 | 73 | AGTTCTTGCTAATGATGGGCCCAAAGTTATATTAAGAACTGCAAAGTAAATTTCAACCAATTACTTTATTCAG | c.8217+32029_8217+32101 | Intronic SNV | c.8217+32103G>T | g.31613687C>A | r.[8217_8218ins8217+32029_8217+32101,=] | Out-of-frame | BMD | [23] |
| 34 | 56 | 166 | AAATATTAAGAATTGTTGACTACAACAGTATGGAAAAGCAATAGATTCCAGTGTGTATTTCATGCCAAAAGTCTCAGCATTCTGCATGTGGAAATAAACATATGGCTAAACACTGCCTTTTCTCAAAATTGCCATCAAACTATCCTCTGTTTTGTGGCTCTCAAAA | c.8391-300_8391-135 | Large and small intronic deletions | c.[8391-917_8391-326del;8391-101_8391-73del] | g.[31515134_31515162del;31515387_31515978del] | r.[8390_8391ins8391-300_8391-135,=] | Out-of-frame | BMD | [27] |
| 35 | 60 | 89 | GTTGAGTCCTCCAAGAAGCAGATGCCAGGGCAGATTTATTGCAGAATAACACCTGTGAAGAAATAGGGGTGGAAGCAGAATTGAACAAG | c.9085-15609_9085-15521 | Intronic SNV | c.9085-15519G>T | g.31382270C>A | r.[9084_9085ins9085-15609_9085-15521,=] | Out-of-frame | IMD/DMD | [1] |
| 36 | 62 | 46 | CTTATCCACATCCATCTTGGTCCCCAAAGCCATTGCATGTCAGATG | c.9224+9144_9224+9189 | Intronic SNV | c.9224+9192C>A | g.31332523G>T | r.9224_9225ins9224+9144_9224+9189 | Out-of-frame | IMD | [28] |
| 37 | 62 | 67 | GAGCTGATAGCCAGCAACCACACTTCAAGAAATGGAAGACAGCTGTGAATGCTTCATTCAGGCCCA(A>G) | c.9225-713_9225-647 | Intronic SNV | c.9225-647A>G | g.31279780T>C | r.[=,9224_9225ins9225-713_9925-647{9225-647a>g}] | Out-of-frame | BMD/DMD | [1,2,29] |
| 38 | 62 | 58 | TGTTCCACATTGGATGGGTGAAGAAGTCCTGATAGTCGATTATTGATCACATAACAAG | c.9225-347_9225-290 | Intronic SNV | c.9225-285A>G | g.31279418T>C | r.[=,9224_9225ins9225-347_9225-290] | Out-of-frame | BMD | [3,15] |
| 39 | 62 | 208 | TTGTCGGTGTCCTTTCTGTAGTGTTCCACATTGGATGGGTGAAGAAGTCCTGATAGTCGATTATTGATCACATAACAAGGTCAATTTATCATAACTGAAGTGCGATCGATTTGTGGGTGCAAAGAAGAAACAAATTCTGGAACCGAATAATGTTTATATTGCTTTTCTCTTTGGAAACAAAGCAGAAAGGGGCCTTTCTGCTTGTAAG | c.9225-368_9225-161 | Intronic SNV | c.9225-160A>G | g.31279293T>C | r.[=,9224_9225ins9225-368_9225-161] | Out-of-frame | BMD | [30] |
| 40 | 65 | 147 | ATGACATGTGAATGCATTCTGAATGTATAACTTCCTTCTACCTGACTGAAAAGTATTTGGTGACAATTTTAACTCCTTGAAGACCTGAGTTGCTGTATAAAGTGGATTTGTTAAATTTTGATCTACCTTTTCTTAAGAGGGAGAAAG | c.9563+1068_9563+1214 | Intronic SNV | c.9563+1215A>G | g.31226400T>C | r.9563_9564ins9563+1068_9563+1214/ r.[=,9563_9564ins9563+1068_9563+1214] | Out-of-frame | DMD | [2,21] |
| 41 | 65 | 53 | GGGCAATCTGATGAAGATCTGAGCATTTAAGAGGGCTGAGCAGTTAGTTGCTG | c.9564-484_9564-432 | Intronic SNV | c.9564-427T>G | g.31225211A>C | r.9563_9564ins9564-484_9564-432 | Out-of-frame | DMD | [6] |
| 42 | 67 | 121 | AAGGGGTCTAACTTCGTCACCCAGGCTGGAGTGCAGTGGCACGATCACAGCTCATTGCAGCCTCGACCTCTGGGCTCAAGTGATCCTCCCACCTCAGCCTCCTGAGTAGCTGGGACTACAG | c.9807+2592_9807+2712 | Intronic SNV | c.9807+2714C>T | g.31219364G>A | r.9807_9808ins9807+2592_9807+2712 | Out-of-frame | DMD | [3] |

PEs and related genetics details were consistently recorded in relation to genomic reference sequence NC_000023.10 (genome build GRCh37/hg19), coding DNA reference sequence NM_004006.2, RNA reference sequence NM_004006.2, and protein reference sequence NP_003997.1 according to the Human Genome Variation Society nomenclature[31], published literature, and Leiden Open Variation Database (https://databases.lovd.nl/shared/variants/DMD/unique). Single-base substitution was shown in the form of “(X > Y),” where “X” was the reference nucleotide and Y was the mutated one. Insertions and deletion-insertions were underlined. Inverted sequence is italicized. DMD, Duchenne muscular dystrophy; IMD, Intermediate muscular dystrophy; BMD, Becker muscular dystrophy; XLDCM, X-linked dilated cardiomyopathy; PE, pseudoexon; SNV, single nucleotide variant; ins, insertion; inv, inversion; dup, duplication; del, deletion; delins, deletion-insertion; NA, unavailable in the published studies.

**References**

1. Béroud, C.; Carrié, A.; Beldjord, C.; Deburgrave, N.; Llense, S.; Carelle, N.; Peccate, C.; Cuisset, J.M.; Pandit, F.; Carré-Pigeon, F.; et al. Dystrophinopathy caused by mid-intronic substitutions activating cryptic exons in the DMD gene. *Neuromuscul. Disord.* **2004**, *14*, 10–18, doi:10.1016/S0960-8966(03)00169-X.
2. Deburgrave, N.; Daoud, F.; Llense, S.; Barbot, J.C.; Récan, D.; Peccate, C.; Burghes, A.H.M.; Béroud, C.; Garcia, L.; Kaplan, J.-C.; et al. Protein- and mRNA-based phenotype-genotype correlations in DMD/BMD with point mutations and molecular basis for BMD with nonsense and frameshift mutations in the DMD gene. *Hum. Mutat.* **2007**, *28*, 183–195, doi:10.1002/humu.20422.
3. Takeshima, Y.; Yagi, M.; Okizuka, Y.; Awano, H.; Zhang, Z.; Yamauchi, Y.; Nishio, H.; Matsuo, M. Mutation spectrum of the dystrophin gene in 442 Duchenne/Becker muscular dystrophy cases from one Japanese referral center. *J. Hum. Genet.* **2010**, *55*, 379–388, doi:10.1038/jhg.2010.49.
4. Yagi, M.; Takeshima, Y.; Wada, H.; Nakamura, H.; Matsuo, M. Two alternative exons can result from activation of the cryptic splice acceptor site deep within intron 2 of the dystrophin gene in a patient with as yet asymptomatic dystrophinopathy. *Hum. Genet.* **2003**, *112*, 164–170, doi:10.1007/s00439-002-0854-8.
5. Xu, Y.; Song, T.; Li, Y.; Guo, F.; Jin, X.; Cheng, L.; Zheng, J.; Li, C.; Zhang, Y.; Chen, B.; et al. Identification of two novel insertion abnormal transcripts in two Chinese families affected with Dystrophinopathy. *J. Clin. Lab. Anal.* **2020**, *34*, 1–6, doi:10.1002/jcla.23142.
6. Sedláčková, J.; Vondráček, P.; Hermanová, M.; Zámečník, J.; Hrubá, Z.; Haberlová, J.; Kraus, J.; Maříková, T.; Hedvičáková, P.; Voháňka, S.; et al. Point mutations in Czech DMD/BMD patients and their phenotypic outcome. *Neuromuscul. Disord.* **2009**, *19*, 749–753, doi:10.1016/j.nmd.2009.08.011.
7. Zaum, A.K.; Stüve, B.; Gehrig, A.; Kölbel, H.; Schara, U.; Kress, W.; Rost, S. Deep intronic variants introduce DMD pseudoexon in patient with muscular dystrophy. *Neuromuscul. Disord.* **2017**, *27*, 631–634, doi:10.1016/j.nmd.2017.04.003.
8. Tuffery-Giraud, S.; Saquet, C.; Thorel, D.; Disset, A.; Rivier, F.; Malcolm, S.; Claustres, M. Mutation spectrum leading to an attenuated phenotype in dystrophinopathies. *Eur. J. Hum. Genet.* **2005**, *13*, 1254–1260, doi:10.1038/sj.ejhg.5201478.
9. Rimessi, P.; Fabris, M.; Bovolenta, M.; Bassi, E.; Falzarano, S.; Gualandi, F.; Rapezzi, C.; Coccolo, F.; Perrone, D.; Medici, A.; et al. Antisense modulation of both exonic and intronic splicing motifs induces skipping of a DMD pseudo-exon responsible for X-linked dilated cardiomyopathy. *Hum. Gene Ther.* **2010**, doi:10.1089/hum.2010.010.
10. Gualandi, F.; Rimessi, P.; Cardazzo, B.; Toffolatti, L.; Dunckley, M.G.; Calzolari, E.; Patarnello, T.; Muntoni, F.; Ferlini, A. Genomic definition of a pure intronic dystrophin deletion responsible for an XLDC splicing mutation: In vitro mimicking and antisense modulation of the splicing abnormality. *Gene* **2003**, *311*, 25–33, doi:10.1016/S0378-1119(03)00527-4.
11. Ferlini, A.; Galié, N.; Merlini, L.; Sewry, C.; Branzi, A.; Muntoni, F. A novel Alu-like element rearranged in the dystrophin gene causes a splicing mutation in a family with X-linked dilated cardiomyopathy. *Am. J. Hum. Genet.* **1998**, *63*, 436–446, doi:10.1086/301952.
12. Gurvich, O.L.; Tuohy, T.M.; Howard, M.T.; Finkel, R.S.; Medne, L.; Anderson, C.B.; Weiss, R.B.; Wilton, S.D.; Flanigan, K.M. DMD pseudoexon mutations: Splicing efficiency, phenotype, and potential therapy. *Ann. Neurol.* **2008**, *63*, 81–89, doi:10.1002/ana.21290.
13. Xie, Z.; Sun, C.; Liu, Y.; Yu, M.; Zheng, Y.; Meng, L.; Wang, G.; Cornejo, D.M.; Bharadwaj, T; Yan, J.; et al. Practical approach to the genetic diagnosis of unsolved dystrophinopathies: A stepwise strategy in the genomic era. *J. Med. Genet*. **2020**, Sep 25, doi:10.1136/jmedgenet-2020-107113.
14. Savarese, M.; Qureshi, T.; Torella, A.; Laine, P.; Giugliano, T.; Jonson, P.H.; Johari, M.; Paulin, L.; Piluso, G.; Auvinen, P.; et al. Identification and Characterization of Splicing Defects by Single-Molecule Real-Time Sequencing Technology (PacBio). *J. Neuromuscul. Dis.* **2020**, 1–5, doi:10.3233/jnd-200523.
15. Tuffery-Giraud, S.; Saquet, C.; Chambert, S.; Claustres, M. Pseudoexon activation in the DMD gene as a novel mechanism for becker muscular dystrophy. *Hum. Mutat.* **2003**, *21*, 608–614, doi:10.1002/humu.10214.
16. Ikezawa, M.; Minami, N.; Takahashi, M.; Goto, Y.I.; Miike, T.; Nonaka, I. Dystrophin gene analysis on 130 patients with Duchenne muscular dystrophy with a special reference to muscle mRNA analysis. *Brain Dev.* **1998**, *20*, 165–168, doi:10.1016/S0387-7604(98)00012-6.
17. Ikezawa, M.; Nishino, I.; Goto, Y.; Miike, T.; Nonaka, I. Newly recognized exons induced by a splicing abnormality from an intronic mutation of the dystrophin gene resulting in Duchenne muscular dystrophy. Mutations in brief no. 213. Online. *Hum. Mutat.* **1999**, *13*, 170, doi:10.1002/(SICI)1098-1004(1999)13:2<170::AID-HUMU12>3.0.CO;2-7.
18. Trabelsi, M.; Beugnet, C.; Deburgrave, N.; Commere, V.; Orhant, L.; Leturcq, F.; Chelly, J. When a mid-intronic variation of DMD gene creates an ESE site. *Neuromuscul. Disord.* **2014**, *24*, 1111–1117, doi:10.1016/j.nmd.2014.07.003.
19. Jin, M.; Li, J.J.; Xu, G.R.; Wang, N.; Wang, Z.Q. Cryptic exon activation causes dystrophinopathy in two Chinese families. *Eur. J. Hum. Genet.* **2020**, doi:10.1038/s41431-020-0578-z.
20. Ishmukhametova, A.; Van Kien, P.K.; Méchin, D.; Thorel, D.; Vincent, M.C.; Rivier, F.; Coubes, C.; Humbertclaude, V.; Claustres, M.; Tuffery-Giraud, S. Comprehensive oligonucleotide array-comparative genomic hybridization analysis: New insights into the molecular pathology of the DMD gene. *Eur. J. Hum. Genet.* **2012**, *20*, 1096–1100, doi:10.1038/ejhg.2012.51.
21. Bovolenta, M.; Neri, M.; Fini, S.; Fabris, M.; Trabanelli, C.; Venturoli, A.; Martoni, E.; Bassi, E.; Spitali, P.; Brioschi, S.; et al. A novel custom high density-comparative genomic hybridization array detects common rearrangements as well as deep intronic mutations in dystrophinopathies. *BMC Genomics* **2008**, *9*, 1–11, doi:10.1186/1471-2164-9-572.
22. Gonorazky, H.; Liang, M.; Cummings, B.; Lek, M.; Micallef, J.; Hawkins, C.; Basran, R.; Cohn, R.; Wilson, M.D.; Macarthur, D.; et al. RNAseq analysis for the diagnosis of muscular dystrophy. *Ann. Clin. Transl. Neurol.* **2016**, *3*, 55–60, doi:10.1002/acn3.267.
23. Cummings, B.B.; Marshall, J.L.; Tukiainen, T.; Lek, M.; Donkervoort, S.; Foley, A.R.; Bolduc, V.; Waddell, L.B.; Sandaradura, S.A.; O’Grady, G.L.; et al. Improving genetic diagnosis in Mendelian disease with transcriptome sequencing. *Sci. Transl. Med.* **2017**, doi:10.1126/scitranslmed.aal5209.
24. Greer, K.; Mizzi, K.; Rice, E.; Kuster, L.; Barrero, R.A.; Bellgard, M.I.; Lynch, B.J.; Foley, A.R.; Rathallaigh, E.O.; Wilton, S.D.; et al. Pseudoexon activation increases phenotype severity in a becker muscular dystrophy patient. *Mol. Genet. Genomic Med.* **2015**, *3*, 320–326, doi:10.1002/mgg3.144.
25. Barreo RA, F.S. Targeted Suppression of a Dystrophin Pseudo-exon using Antisense Oligonucleotides. *J. Genet. Syndr. Gene Ther.* **2014**, *05*, doi:10.4172/2157-7412.1000235.
26. Gonçalves, A.; Oliveira, J.; Coelho, T.; Taipa, R.; Melo-Pires, M.; Sousa, M.; Santos, R. Exonization of an intronic LINE-1 element causing becker muscular dystrophy as a novel mutational mechanism in dystrophin gene. *Genes (Basel).* **2017**, *8*, 1–9, doi:10.3390/genes8100253.

27. Khelifi, M.M.; Ishmukhametova, A.; Van Kien, P.K.; Thorel, D.; Méchin, D.; Perelman, S.; Pouget, J.; Claustres, M.; Tuffery-Giraud, S. Pure intronic rearrangements leading to aberrant pseudoexon inclusion in dystrophinopathy: A new class of mutations? *Hum. Mutat.* **2011**, *32*, 467–475, doi:10.1002/humu.21471.
28. Santos, R.; Gonçalves, A.; Oliveira, J.; Vieira, E.; Vieira, J.P.; Evangelista, T.; Moreno, T.; Santos, M.; Fineza, I.; Bronze-Da-Rocha, E. New variants, challenges and pitfalls in DMD genotyping: Implications in diagnosis, prognosis and therapy. *J. Hum. Genet.* **2014**, *59*, 454–464, doi:10.1038/jhg.2014.54.
29. Juan-Mateu, J.; González-Quereda, L.; Rodríguez, M.J.; Verdura, E.; Lázaro, K.; Jou, C.; Nascimento, A.; Jiménez-Mallebrera, C.; Colomer, J.; Monges, S.; et al. Interplay between DMD Point Mutations and Splicing Signals in Dystrophinopathy Phenotypes. *PLoS One* **2013**, *8*, doi:10.1371/journal.pone.0059916.
30. Daoud, F.; Angeard, N.; Demerre, B.; Martie, I.; Benyaou, R.; Leturcq, F.; Cossée, M.; Deburgrave, N.; Saillour, Y.; Tuffery, S.; et al. Analysis of Dp71 contribution in the severity of mental retardation through comparison of Duchenne and Becker patients differing by mutation consequences on Dp71 expression. *Hum. Mol. Genet.* **2009**, *18*, 3779–3794, doi:10.1093/hmg/ddp320.
31. den Dunnen, J.T.; Dalgleish, R.; Maglott, D.R.; Hart, R.K.; Greenblatt, M.S.; Mcgowan-Jordan, J.; Roux, A.F.; Smith, T.; Antonarakis, S.E.; Taschner, P.E.M. HGVS Recommendations for the Description of Sequence Variants: 2016 Update. *Hum. Mutat.* **2016**, *37*, 564–569, doi:10.1002/humu.22981.
