# Supplementary material for: Splicing Characteristics of Dystrophin Pseudoexons and Identification of a Novel Pathogenic Intronic Variant in the DMD Gene
Source: Genes (Basel). 2020 Oct 10;11(10):1180. doi: 10.3390/genes11101180 (PMC7650627; doi:10.3390/genes11101180)
Supplement: Supplementary file 1 [file genes-11-01180-s001.zip › Supplementary files/Table S3.pdf]

Table S3. Statistics data of each essential splicing signal and splicing regulatory element of different groups of dystrophin pseudoexons and the group of dystrophin canonical exons.

	Dystrophin CEs (77)		Splice site group (31)		SRE group (11)		Poison exon group (5)		Total PEs (42)		Kruskal-Wallis test	Nemenyi test						Mann-Whitney U test
	Median	(25th, 75th)	Median	(25th, 75th)	Median	(25th, 75th)	Median	(25th, 75th)	Median	(25th, 75th)		CEs vs splice site group	CEs vs SRE group	CEs vs poison exon group	Splice site group vs SRE group	Splice site group vs poison exon group	SRE group vs poison exon group	CEs vs total PEs
**Exon size (bp)**	**150**	**(110, 176)**	95	(72, 137)	159	(77, 167)	**67**	**(48, 75)**	**96**	**(73, 159)**	**< 0.001**	0.135	0.887	**< 0.001**	0.401	0.936	**< 0.001**	**0.002**
5′ ss strength (HSF)	88.630	(84.145, 90.255)	89.780	(82.570, 93.690)	83.820	(81.810, 89.780)	90.440	(82.595, 95.630)	88.390	(82.235, 92.838)	0.235	–	–	–	–	–	–	0.728
5′ ss strength (MaxEnt)	8.550	(7.095, 9.460)	8.830	(8.170, 10.360)	7.960	(7.400, 9.720)	8.760	(6.770, 10.840)	8.720	(7.938, 10.135)	0.165	–	–	–	–	–	–	0.145
5′ ss strength (MDD)	12.680	(10.780, 13.930)	13.280	(11.980, 14.880)	11.780	(10.380, 13.880)	13.880	(9.930, 15.180)	12.980	(11.455, 14.580)	0.204	–	–	–	–	–	–	0.321
5′ ss strength (MM)	7.280	(6.500, 8.400)	8.280	(7.040, 10.100)	6.310	(5.650, 8.510)	8.280	(5.570, 10.985)	7.980	(6.310, 9.115)	0.160	–	–	–	–	–	–	0.314
5′ ss strength (WMM)	7.910	(6.510, 8.975)	8.800	(6.180, 10.360)	7.400	(4.880, 9.290)	9.620	(5.940, 11.180)	8.110	(5.655, 9.620)	0.259	–	–	–	–	–	–	0.859
**3′ ss strength (HSF)**	86.820	(82.800, 90.710)	85.890	(82.000, 91.060)	**84.710**	**(81.530, 89.290)**	**90.780**	**(88.720, 92.285)**	85.235	(81.985, 89.888)	**0.021**	0.923	0.653	0.992	0.476	0.948	**0.002**	0.409
**3′ ss strength (MaxEnt)**	8.430	(6.340, 9.805)	8.180	(6.490, 10.320)	**7.490**	**(5.980, 8.190)**	**10.550**	**(9.145, 11.560)**	8.060	(6.410, 9.990)	**0.009**	0.877	0.676	0.504	0.633	0.363	**< 0.001**	0.395
**3′ ss strength (MM)**	**9.110**	**(6.870, 10.410)**	**9.290**	**(5.580, 11.280)**	**7.720**	**(6.800, 9.130)**	**11.360**	**(10.180, 12.565)**	8.515	(5.663, 10.460)	**0.043**	0.776	0.121	**0.006**	0.067	**0.013**	**< 0.001**	0.543
3′ ss strength (WMM)	8.440	(6.260, 11.415)	8.740	(6.130, 11.150)	8.710	(7.820, 10.110)	11.040	(8.880, 15.310)	8.740	(6.393, 10.928)	0.328	–	–	–	–	–	–	0.905
BP distance to 3' ss (bp)	28	(23, 34)	28.000	(24, 37)	29	(23, 39)	32	(25, 40)	2	(23, 37)	0.800	–	–	–	–	–	–	0.713
Pyrimidine content*	0.722	(0.663, 0.814)	0.684	(0.619, 0.741)	0.719	(0.706, 0.818)	0.765	(0.532, 0.863)	0.712	(0.641, 0.769)	0.560	–	–	–	–	–	–	0.333
**ESE density (RESCUE-ESE)**	**0.167**	**(0.117, 0.208)**	0.115	(0.076, 0.192)	**0.084**	**(0.027, 0.121)**	**0.082**	**(0.011, 0.140)**	**0.111**	**(0.048, 0.156)**	**< 0.001**	0.722	**< 0.001**	**0.002**	0.221	0.373	0.464	**< 0.001**
ESE density (PESE)	0.078	(0.049, 0.102)	0.072	(0.022, 0.101)	0.056	(0.012, 0.065)	0.022	(0.006, 0.074)	0.060	(0.022, 0.098)	0.621	–	–	–	–	–	–	0.058
ESE density (SRp55)	0.017	(0.011, 0.028)	0.020	(0.011, 0.033)	0.014	(0.012, 0.034)	0.020	(0.007, 0.063)	0.020	(0.012, 0.033)	0.891	–	–	–	–	–	–	0.570
ESE density (SRp40)	0.041	(0.030, 0.054)	0.034	(0.025, 0.038)	0.048	(0.036, 0.056)	0.026	(0.017, 0.052)	0.036	(0.029, 0.042)	0.672	–	–	–	–	–	–	0.066
ESE density (SC35)	0.032	(0.022, 0.044)	0.032	(0.015, 0.047)	0.036	(0.026, 0.050)	0.026	(0.007, 0.046)	0.035	(0.015, 0.047)	0.697	–	–	–	–	–	–	0.967
**ESE density (ASFB)**	0.033	(0.026, 0.046)	0.023	(0.014, 0.038)	**0.043**	**(0.026, 0.058)**	**0.020**	**(0.000, 0.034)**	0.027	(0.018, 0.045)	**0.007**	0.982	0.399	0.596	0.568	0.323	**0.006**	0.070
ESE density (ASF)	0.037	(0.021, 0.046)	0.029	(0.020, 0.042)	0.031	(0.026, 0.050)	0.020	(0.000, 0.034)	0.030	(0.020, 0.044)	0.245	–	–	–	–	–	–	0.617
**ESE density (Tra2)**	**0.033**	**(0.022, 0.057)**	0.030	(0.014, 0.053)	0.026	(0.000, 0.060)	**0.000**	**(0.000, 0.022)**	0.030	(0.011, 0.053)	**0.014**	0.127	0.091	**< 0.001**	0.611	0.169	0.998	0.071
**ESE density (9G8)**	**0.099**	**(0.081, 0.112)**	0.072	(0.059, 0.113)	**0.063**	**(0.037, 0.095)**	**0.041**	**(0.020, 0.070)**	**0.067**	**(0.050, 0.104)**	**0.001**	0.611	**< 0.001**	**< 0.001**	0.769	0.472	0.514	**0.004**
**ESE density (EIE)**	**0.410**	**(0.348, 0.456)**	0.308	(0.234, 0.411)	**0.308**	**(0.171, 0.401)**	**0.219**	**(0.155, 0.343)**	**0.308**	**(0.231, 0.411)**	**< 0.001**	0.685	**0.002**	**< 0.001**	0.912	0.433	0.764	**< 0.001**
**ESE density (NI-ESE)**	**0.415**	**(0.370, 0.450)**	**0.347**	**(0.279, 0.386)**	**0.280**	**(0.230, 0.299)**	**0.235**	**(0.150, 0.350)**	**0.310**	**(0.252, 0.378)**	**< 0.001**	**< 0.001**	**< 0.001**	**< 0.001**	0.657	0.122	0.388	**< 0.001**
ESS density (Sironi)	0.125	(0.108, 0.147)	0.120	(0.089, 0.151)	0.105	(0.081, 0.150)	0.078	(0.063, 0.120)	0.113	(0.089, 0.150)	0.139	–	–	–	–	–	–	0.368
ESS density (PESS)	0.011	(0.005, 0.024)	0.011	(0.000, 0.031)	0.018	(0.012, 0.038)	0.000	(0.000, 0.036)	0.014	(0.000, 0.035)	0.208	–	–	–	–	–	–	0.622
**ESS density (IIE)**	**0.150**	**(0.113, 0.211)**	**0.182**	**(0.134, 0.286)**	**0.176**	**(0.170, 0.241)**	**0.192**	**(0.131, 0.314)**	**0.181**	**(0.152, 0.274)**	**0.010**	**< 0.001**	**0.009**	**0.003**	0.233	0.312	0.766	**0.005**
**ESS density (FAS)**	**0.018**	**(0.010, 0.033)**	**0.033**	**(0.010, 0.041)**	**0.024**	**(0.006, 0.041)**	0.026	(0.000, 0.033)	**0.029**	**(0.009, 0.041)**	**0.025**	**0.033**	**0.041**	0.821	0.901	0.333	0.549	**0.035**
ESS density (hnRNPA1)	0.059	(0.042, 0.075)	0.052	(0.039, 0.078)	0.050	(0.030, 0.076)	0.041	(0.010, 0.055)	0.052	(0.037, 0.076)	0.188	–	–	–	–	–	–	0.326
**ESS density (NI-ESS)**	**0.077**	**(0.057, 0.110)**	**0.130**	**(0.061, 0.168)**	**0.135**	**(0.087, 0.163)**	**0.154**	**(0.048, 0.264)**	**0.131**	**(0.066, 0.163)**	**0.020**	**0.002**	**0.007**	**< 0.001**	0.870	0.090	0.466	**0.003**
**Total ESEs density**	**1.392**	**(1.218, 1.510)**	**1.144**	**(0.947, 1.370)**	**0.981**	**(0.780, 1.180)**	**0.627**	**(0.545, 1.097)**	**1.123**	**(0.831, 1.288)**	**< 0.001**	**0.008**	**< 0.001**	**< 0.001**	0.432	0.087	0.322	**< 0.001**
**Total ESSs density**	**0.438**	**(0.404, 0.554)**	**0.585**	**(0.410, 0.658)**	**0.564**	**(0.447, 0.629)**	**0.410**	**(0.346, 0.770)**	**0.568**	**(0.433, 0.656)**	**0.009**	**0.006**	**0.017**	**0.015**	0.376	0.401	0.477	**0.003**
**ESEs/ESSs ratio (total)**	**2.886**	**(2.443, 3.506)**	**2.079**	**(1.420, 2.685)**	**1.569**	**(1.391, 2.274)**	**1.438**	**(0.803, 2.979)**	**2.050**	**(1.413, 2.683)**	**< 0.001**	**< 0.001**	**< 0.001**	**0.002**	0.071	0.341	0.676	**< 0.001**
**ISSs density (3′ ss)**	**0.187**	**(0.152, 0.227)**	**0.163**	**(0.140, 0.190)**	0.177	(0.153, 0.197)	**0.163**	**(0.125, 0.217)**	**0.167**	**(0.143, 0.192)**	**0.029**	**0.038**	0.760	**0.040**	0.665	0.901	0.812	**0.031**
ISEs density (3′ ss)	0.452	(0.358, 0.577)	0.407	(0.283, 0.553)	0.470	(0.293, 0.540)	0.407	(0.297, 0.603)	0.415	(0.292, 0.543)	0.447	–	–	–	–	–	–	0.187
ISSs/ISEs ratio (3′ ss)	0.398	(0.343, 0.477)	0.395	(0.314, 0.589)	0.418	(0.327, 0.512)	0.395	(0.255, 0.654)	0.405	(0.321, 0.573)	0.983	–	–	–	–	–	–	0.965
ISSs density (5′ ss)	0.170	(0.143, 0.207)	0.173	(0.147, 0.213)	0.177	(0.130, 0.283)	0.193	(0.143, 0.245)	0.173	(0.145, 0.213)	0.966	–	–	–	–	–	–	0.918
ISEs density (5′ ss)	0.377	(0.338, 0.543)	0.450	(0.313, 0.660)	0.460	(0.367, 0.550)	0.450	(0.358, 0.588)	0.455	(0.351, 0.628)	0.523	–	–	–	–	–	–	0.172
ISSs/ISEs ratio (5′ ss)	0.430	(0.344, 0.517)	0.377	(0.286, 0.538)	0.358	(0.320, 0.484)	0.374	(0.337, 0.556)	0.375	(0.305, 0.538)	0.762	–	–	–	–	–	–	0.326

The nonparametric Kruskal-Wallis test was used to compare the difference in each splicing signal among different groups of dystrophin PEs and the group of dystrophin CEs. If Kruskal-Wallis test was statistically significant, the Nemenyi test was used to perform pairwise comparisons to locate the source of significance. The Mann-Whitney U test was employed to compare the difference in each splicing signal between the group of total dystrophin PEs and the group of dystrophin CEs. A splicing signal with significant difference among different groups was bolded. *, The pyrimidine content between the BP adenine and the 3' ss were calculated. CEs, dystrophin canonical exons; Splice site group, dystrophin pseudoexons with alterations in splice sites; SRE group, dystrophin pseudoexons with alterations in splicing regulatory elements; Poison exon group, dystrophin pseudoexons with the characteristics of poison exons; total PEs, total dystrophin pseudoexons; 25th, the 25th percentile; 75th, the 75th percentile; HSF, Human Splicing Finder; MaxEnt, maximum entropy; MDD, multiple dependence decomposition; MM, first order Markov model; WMM, weight matrix model; BP, branch point; ss, splice site; SD, standard deviation; ESE, exonic splicing enhancers; ESS, exonic splicing silencers; ISE, intronic splicing enhancers; ISS, intronic splicing silencers; EIE, exon-identity element; IIE, intron-identity element; NI, neighbourhood inference.
