# Supplementary material for: Splicing Characteristics of Dystrophin Pseudoexons and Identification of a Novel Pathogenic Intronic Variant in the DMD Gene
Source: Genes (Basel). 2020 Oct 10;11(10):1180. doi: 10.3390/genes11101180 (PMC7650627; doi:10.3390/genes11101180)
Supplement: Supplementary file 1 [file genes-11-01180-s001.zip › Supplementary files/Table S1.pdf]

Table S1. The list of primers used for the *DMD* gene cDNA sequence amplification.

Amplified fragment (range)	Size (bp)	Forward primer sequence (5′ -> 3′)	Reverse primer sequence (5′ -> 3′)
F1 (exons 1~ 7)	606	ACTGGAGCAATAAAGTTTGAAGAAC	CTGGCCTATGACTATGGATGAGA
F2 (exons 6~10)	597	AAGATTCTCCTGAGCTGGGTC	CTCCATCAATGAACTGCCAAA
F3 (exons 9~13)	649	GGCTGCTTATGTCACCACCTCT	TTCATCAACTACCACCACCATG
F4 (exons 12~17)	686	AGTACAACAACATAAGGTGCTTCA	AATCCACAGTAATCTGCCTCTTC
F5 (exons 17~21)	631	CACCACTCAGCCATCACTAACA	TAGCCGGTTGACTTCATCCTTA
F6 (exons 20~24)	725	CAGAACAACATCATCGCTTTCTAT	AAAACATCAACTTCAGCCATCC
F7 (exons 23~27)	618	AAATTGAGGGACGCTGGAA	GTGGAGCTTGAGCTATGACACTA
F8 (exons 27~30)	491	CCAAACTCTAACCACCAACTACCA	AGCTGCGTCCACCTTGTCTG
F8_2 (exons 26~30)	701	CTGTAAGCCTCCAGAAAGAT	CTGCTTGTCAATGAATGTGA
F9 (exons 30~34)	696	GAATCCAGGAGTCCCTCACAT	CAGGCAACTTCAGAATCCAAA
F10 (exons 34~37)	567	ATTGTCCCGTAAGATGCGAAAG	AGCTCTGAGATTTGGGGCTCTA
F11 (exons 37~41)	625	ATACGCCCAAAGGTGGACTC	CCTCAGCTTGCCTACGCACT
F12 (exons 40~44)	606	CAAATTAGCCAGCCTACCTG	GTCAAATCGCCCTTGTCG
F13 (exons 43~47)	671	TATTCATAGCAAGAAGACAGCAGCAT	GCACGGGTCCTCCAGTTTCA
F14 (exons 46~50)	592	GATAACATTGCTAGTATCCCACTT	CTAGGTCAGGCTGCTTTGC
F15 (exons 49~53)	527	AAAACCAGCCACTCAGCC	TGGTGTTCTTGTACTTCATCCC
F16 (exons 52~55)	571	AAAACAAGACCAGCAATCAAG	GAGTCTTCTAGGAGCCTTTCC
F17 (exons 55~59)	632	AAGTTTCTTGCCTGGCTTACA	TCCTCAGCCTGCTTTCGTAG
F18 (exons 58~63)	623	GACAGAGCAGCCTTTGGAAG	TTGTTTGAGTCTCGTGGTTGATA
F18_2 (exons 58~65)	859	GCAGCCTTTGGAAGGACTA	CAGGCGGTCATAAATAGTGG
F19 (exons 61~67)	627	GGACTTTGGTCCAGCATCTCA	GCAACTTCACCCAACTGTCTTG
F19_2 (exons 60~67)	791	CAGCTTACCACTTTGGGCATTC	CGGACACTTGGCTCAATGTTAC
F20 (exons 65~70)	633	CGGGACGAACAGGGAGGAT	TCTGCACTGGCAGGTAGCC
F21 (exons 69~75)	628	TTGCACTCCGACTACATCAGG	GTGTTGACGCAGTAGCTTGG
F21_2 (exons 74~79)	782	GATGATGAACATTTGTTAATCCAG	AAAACCATGCGGGAATCA
F22 (exons 75~79)	509	CTAAAGCAGCAGCACGAACA	CAAATCATCTGCCATGTGGA
F22_2 (exons 75~79)	691	CCGTCTAAAGCAGCAGCAC	AGTATAATACCACTACCCTTCACAA
