# Supplementary material for: Splicing Characteristics of Dystrophin Pseudoexons and Identification of a Novel Pathogenic Intronic Variant in the DMD Gene
Source: Genes (Basel). 2020 Oct 10;11(10):1180. doi: 10.3390/genes11101180 (PMC7650627; doi:10.3390/genes11101180)
Supplement: Supplementary file 1 [file genes-11-01180-s001.zip › Supplementary files/Table S4.pdf]

Table S4. Comparative analysis of GERP score of each position in the acceptor splice site consensus motif between dystrophin canonical exons and pseudoexons.

Position	Dystrophin canonical exons	Dystrophin pseudoexons	*P*-value
	Median (range)	Median (range)	
+1	5.175 (0.188 – 6.160)	1.555 (-6.250 – 5.700)	**< 0.001**
-1	5.395 (4.270 – 6.160)	-0.003 (-6.490 – 5.700)	**< 0.001**
-2	5.415 (4.270 – 6.160)	0.428 (-6.250 – 5.700)	**< 0.001**
-3	3.340 (-2.540 – 6.020)	0.246 (-9.610 – 4.830)	**< 0.001**
-4	1.985 (-10.600 – 5.820)	0.317 (-5.130 – 5.700)	**0.003**
-5	3.610 (-3.130 – 6.160)	0.234 (-6.580 – 5.700)	**< 0.001**
-6	3.540 (-5.120 – 6.160)	0.834 (-5.670 – 5.700)	**< 0.001**
-7	2.785 (-5.470 – 6.020)	0.740 (-6.250 – 5.700)	**0.001**
-8	2.480 (-5.290 – 5.820)	0.330 (-7.480 – 5.700)	**< 0.001**
-9	2.990 (-11.300 – 6.020)	0.625 (-6.890 – 4.540)	**< 0.001**
-10	2.710 (-5.090 – 6.160)	0.649 (-6.250 – 5.700)	**0.001**
-11	1.965 (-5.880 – 5.990)	0.580 (-4.070 – 5.700)	**0.035**
-12	3.120 (-9.850 – 6.160)	0.926 (-5.190 – 5.700)	**< 0.001**
-13	2.580 (-11.500 – 6.020)	0.671 (-8.690 – 5.700)	**0.045**
-14	1.910 (-10.200 – 6.160)	0.513 (-8.320 – 5.700)	**0.043**
-15	1.775 (-9.830 – 6.020)	0.381 (-6.840 – 5.000)	**0.049**
-16	2.370 (-9.880 – 5.760)	0.499 (-5.790 – 5.700)	0.064
-17	1.950 (-7.790 – 6.160)	0.197 (-6.840 – 5.700)	**0.002**
-18	1.910 (-9.830 – 6.020)	0.861 (-6.490 – 5.700)	**0.036**
-19	1.985 (-9.390 – 5.760)	0.197 (-8.360 – 5.000)	**0.011**
-20	2.000 (-7.740 – 5.760)	0.527 (-3.590 – 5.700)	0.070

-21	2.200 (-10.200 – 6.020)	1.115 (-7.540 – 5.700)	0.190
-22	2.225 (-10.100 – 6.020)	0.274 (-7.690 – 5.700)	0.058

The Genomic Evolutionary Rate Profiling (GERP) score is calculated based on an alignment of 35 mammalian species. A positive GERP score indicates that a site is probably under evolutionary constraint, whereas a negative GERP score indicates that a site is probably evolving neutrally. A GERP score above the threshold of 2 indicates a highly conserved site.
