# Supplementary material for: Splicing Characteristics of Dystrophin Pseudoexons and Identification of a Novel Pathogenic Intronic Variant in the DMD Gene
Source: Genes (Basel). 2020 Oct 10;11(10):1180. doi: 10.3390/genes11101180 (PMC7650627; doi:10.3390/genes11101180)
Supplement: Supplementary file 1 [file genes-11-01180-s001.zip › Supplementary files/Table S5.pdf]

Table S5. Comparative analyses of essential splicing signals between different splice site groups.

Essential splicing signals	Group I	Group II	*P*-value
**5′ ss strength (HSF)**	**93.13 (89.78, 96.21)**	**82.57 (80.43, 89.02)**	**< 0.001**
**5′ ss strength (MaxEnt)**	**10.06 (8.68, 10.65)**	**8.50 (5.89, 8.83)**	**0.001**
**5′ ss strength (MDD)**	**14.58 (13.18, 15.48)**	**11.98 (10.38, 12.88)**	**0.001**
**5′ ss strength (MM)**	**9.09 (8.28, 10.55)**	**7.13 (6.09, 7.55)**	**< 0.001**
**5′ ss strength (WMM)**	**9.62 (8.28, 11.04)**	**6.18 (4.73, 7.58)**	**< 0.001**
3′ ss strength (HSF)	85.89 (84.37, 89.49)	86.56 (81.94, 91.92)	0.900
3′ ss strength (MaxEnt)	9.59 (7.43, 10.36)	8.15 (6.17, 10.17)	0.395
3′ ss strength (MM)	9.68 (7.22, 11.28)	9.29 (5.23, 11.29)	0.800
3′ ss strength (WMM)	10.03 (5.28, 12.17)	8.74 (6.48, 11.04)	0.582
BP distance to 3' ss (bp)	31.00 (24.00, 44.00)	26 (22, 36)	0.307
Pyrimidine content*	0.72 (0.62, 0.78)	0.68 (0.62, 0.74)	0.525

I: refers to a 5′ ss or 3′ ss that was formed *de novo* or strengthened by a pathogenic variant. II: refers to a 5′ ss or 3′ ss that was only activated as a partner of a mutated splice site. *, The pyrimidine content between the BP adenine and the 3' ss were calculated and compared between the two groups. Descriptive statistics were presented as median (25th percentile, 75th percentile). HSF, Human Splicing Finder; MaxEnt, maximum entropy; MDD, multiple dependence decomposition; MM, first order Markov model; WMM, weight matrix model; BP, branch point; ss, splice site.
