# Supplementary material for: Splicing Characteristics of Dystrophin Pseudoexons and Identification of a Novel Pathogenic Intronic Variant in the DMD Gene
Source: Genes (Basel). 2020 Oct 10;11(10):1180. doi: 10.3390/genes11101180 (PMC7650627; doi:10.3390/genes11101180)
Supplement: Supplementary file 1 [file genes-11-01180-s001.zip › Supplementary files/Table S6.pdf]

Table S6. Comparative analyses of essential splicing signals between *de novo* and canonical splice site groups.

Essential splicing signals	*De novo* group	Canonical group	*P*-value
**5′ ss strength (HSF)**	**93.13 (89.78, 96.21)**	**88.63 (84.15, 90.26)**	**0.004**
**5′ ss strength (MaxEnt)**	**10.06 (8.68, 10.65)**	**8.55 (7.10, 9.46)**	**< 0.001**
**5′ ss strength (MDD)**	**14.58 (13.18, 15.48)**	**12.68 (10.78, 13.93)**	**0.001**
**5′ ss strength (MM)**	**9.09 (8.28, 10.55)**	**7.28 (6.50, 8.40)**	**0.001**
**5′ ss strength (WMM)**	**9.62 (8.28, 11.04)**	**7.91 (6.51, 8.98)**	**0.001**
3′ ss strength (HSF)	85.89 (84.37, 89.49)	86.82 (82.80, 90.71)	0.925
3′ ss strength (MaxEnt)	9.59 (7.43, 10.36)	8.43 (6.34, 9.81)	0.472
3′ ss strength (MM)	9.68 (7.22, 11.28)	9.11 (6.87, 10.41)	0.583
3′ ss strength (WMM)	10.03 (5.28, 12.17)	8.44 (6.26, 11.42)	0.570
BP distance to 3' ss (bp)	31 (24, 44)	28 (23, 34)	0.294
Pyrimidine content*	0.72 (0.62, 0.78)	0.72 (0.66, 0.81)	0.705

The *de novo* group consists of 5′ splice sites or 3′ splice sites that were formed *de novo* or strengthened by a pathogenic variant. The canonical group consists of 5′ splice sites or 3′ splice sites of dystrophin canonical exons. *, The pyrimidine content between the BP adenine and the 3' ss were calculated and compared between the two groups. Descriptive statistics were presented as median (25th percentile, 75th percentile). HSF, Human Splicing Finder; MaxEnt, maximum entropy; MDD, multiple dependence decomposition; MM, first order Markov model; WMM, weight matrix model; BP, branch point; ss, splice site.
