# Supplementary material for: Splicing Characteristics of Dystrophin Pseudoexons and Identification of a Novel Pathogenic Intronic Variant in the DMD Gene
Source: Genes (Basel). 2020 Oct 10;11(10):1180. doi: 10.3390/genes11101180 (PMC7650627; doi:10.3390/genes11101180)
Supplement: Supplementary file 1 [file genes-11-01180-s001.zip › Supplementary files/Figure S1.pdf]

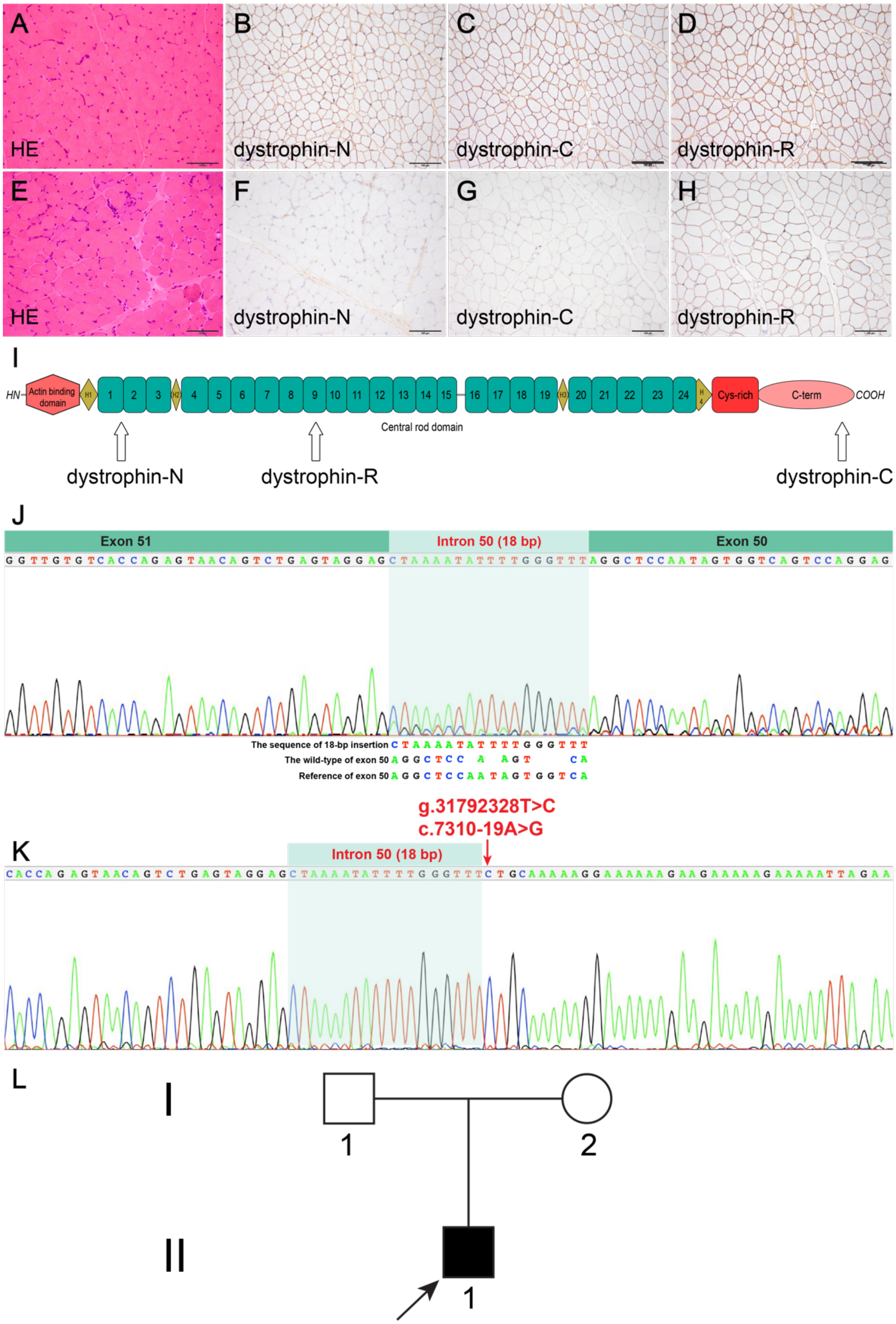
A
HE
B
dystrophin-N
C
dystrophin-C
D
dystrophin-R
E
HE
F
dystrophin-N
G
dystrophin-C
H
dystrophin-R
I
HN
Actin binding domain
H1
1 2 3
H2
4 5 6 7 8 9 10 11 12 13 14 15
16 17 18 19
H3
20 21 22 23 24
H4
Cys-rich
C-term
COOH
Central rod domain
dystrophin-N
dystrophin-R
dystrophin-C
J
Exon 51
Intron 50 (18 bp)
Exon 50
The sequence of 18-bp insertion CTAAAATATTTTGGGTTT
The wild-type of exon 50 AGGCTCC A AGT CA
Reference of exon 50 AGGCTCCAATAGTGGTCA
g.31792328T>C
c.7310-19A>G
K
Intron 50 (18 bp)
L
I
1
2
II
1

Figure S1. Pathological changes of the patient and Sanger sequencing of the aberrant transcript and the genomic variant in the *DMD* gene. (**A**) and (**E**) Hematoxylin and eosin staining (×20); (**B**) and (**F**) Immunohistochemical staining for dystrophin-N (×20); (**C**) and (**G**) dystrophin-C (×20); (**D**) and (**H**) dystrophin-R (×20); (**A**)–(**D**), a healthy control; (**E**)–(**H**), the patient. (**I**) Diagrammatic representation of the dystrophin protein showing four main domains and the positions of the epitopes of the dystrophin-N (amino-terminal), dystrophin-C (carboxyl-terminal), and dystrophin-R (central rod) antibodies. The obvious or partial expression of dystrophin-C regardless of the expression of dystrophin-N and dystrophin-R indicates a molecular diagnosis of Becker muscular dystrophy [1]. (**J**) Sanger sequencing of the aberrant transcript of *DMD* (NM_004006.2) revealed an insertion of 18-bp sequence originating from intron 50 into the mature mRNA between exons 50 and 51. Two overlapping sequences can be recognized following the sequence of exon 51; they can be distinguished into the sequence of exon 50 and the inserted sequence. (**K**) Sanger sequencing of the genomic DNA derived from peripheral blood sample corresponding to the area surrounding the inserted sequence revealed a single-base substitution, g.31792328T>C (c.7310-19A>G), adjacent to the insertion. (**L**) Pedigree of the patient's family. As shown in the pedigree chart, the patient (II:1) had the genomic *DMD* variant (c.7310-19A>G), whereas his parents (unaffected individuals, I:1 and I:2) did not have the variant.

Reference

1. Arahata K, Beggs AH, Honda H, *et al*. Preservation of the C-terminus of dystrophin molecule in the skeletal muscle from Becker muscular dystrophy. *J Neurol Sci*. 1991;101(2):148-156. doi:10.1016/0022-510X(91)90039-A
