# Supplementary material for: Splicing Characteristics of Dystrophin Pseudoexons and Identification of a Novel Pathogenic Intronic Variant in the DMD Gene
Source: Genes (Basel). 2020 Oct 10;11(10):1180. doi: 10.3390/genes11101180 (PMC7650627; doi:10.3390/genes11101180)
Supplement: Supplementary file 1 [file genes-11-01180-s001.zip › Supplementary files/Figure S2.pdf]

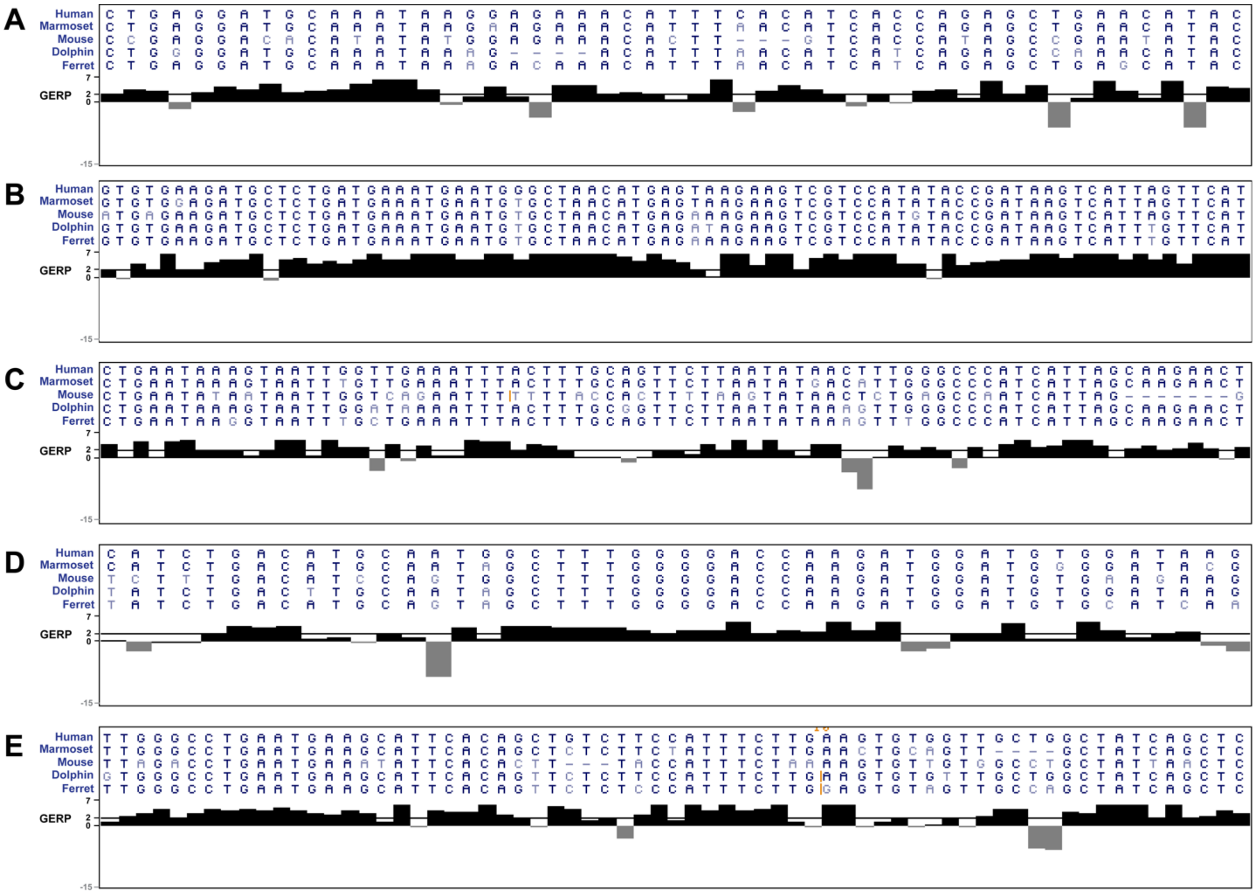

Figure S2. The high-level conservation of five dystrophin pseudoexons. Five dystrophin pseudoexons (PEs) are conserved across representative placental mammals, including Human, Marmoset, Mouse, Ferret, and Dolphin. The high-level conservation of each PE is also illustrated by the Genomic Evolutionary Rate Profiling (GERP) score across the PE region. GERP scores are calculated based on an alignment of 35 mammalian species. A positive GERP score indicates that a site is probably under evolutionary constraint, whereas a negative GERP score indicates that a site is probably evolving neutrally. A GERP score above the threshold of 2 indicates a highly conserved site. These figures were modified from the UCSC Browser. **(A)** PE22 (median GERP score 2.63, range -6.23–5.58); **(B)** PE23 (median GERP score 4.69, range -0.60–5.70); **(C)** PE33 (median GERP score 2.20, range -7.65–4.38); **(D)** PE36 (median GERP score 2.07, range -8.81–4.63); **(E)** PE37 (median GERP score 3.19, range -5.80–5.00).
