# Supplementary material for: Splicing Characteristics of Dystrophin Pseudoexons and Identification of a Novel Pathogenic Intronic Variant in the DMD Gene
Source: Genes (Basel). 2020 Oct 10;11(10):1180. doi: 10.3390/genes11101180 (PMC7650627; doi:10.3390/genes11101180)
Supplement: Supplementary file 1 [file genes-11-01180-s001.zip › Supplementary files/Figure S3.pdf]

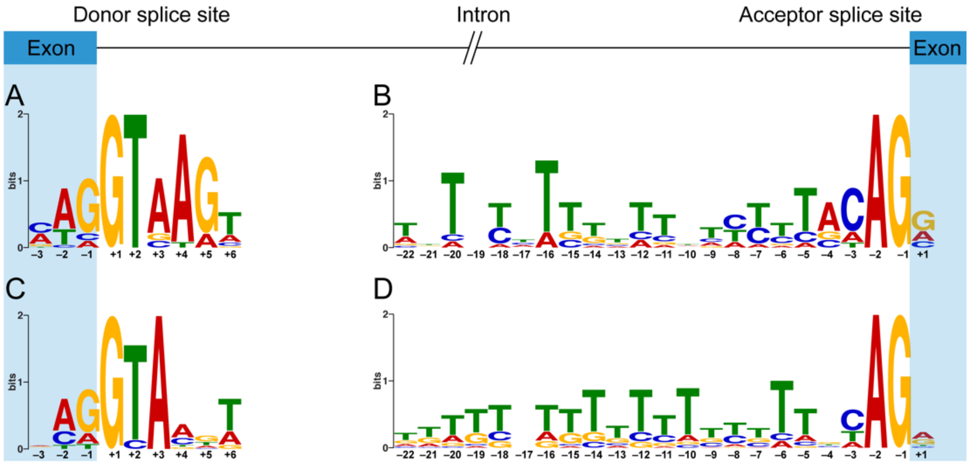

Figure S3. Position weight matrix-based splice site consensus motifs of different splice site groups. Sequence logos for (**A**) the 5′ ss group that were formed *de novo* or strengthened by a pathogenic variant (*de novo* 5′ ss group) and (**C**) the cryptic 5′ ss group that were only activated as partners of a mutated splice site (cryptic 5′ ss group) and the consensus sequences are MAG|GTAAGT and NAG|GTAMKT respectively. Sequence logos for (**B**) the *de novo* 3′ ss group and (**D**) the cryptic 3′ ss group and the consensus sequences are WNTNYHTTTTTTNTYYYTAYAG|R and TKTKTNTTTTTTWTYTYTBYAG|R respectively. The height of each letter reflects the relative frequency of that nucleotide in the respective position. "|" indicates the exon-intron boundary in the consensus sequence. N stands for any nucleotide, H for any nucleotide except G, B for any nucleotide except A, Y for C or T, R for A or G, M for A or C, K for G or T, and W for A or T.
